# Joint changes in subjective memory and objective cognition and risk of incident functional dependence: A harmonized analysis of four aging cohorts

**DOI:** 10.1002/alz.71752

**Published:** 2026-08-22

**Authors:** Xulong Yin, Zhen Li, Rong Fu, Ting Hong, Yi You, Yusheng Zhao, Huiru Shen, Hui Wang, Qi Fang

**Affiliations:** ^1^ Department of Neurology The First Affiliated Hospital of Soochow University Suzhou China; ^2^ Institute of Stroke Research Soochow University Suzhou China

**Keywords:** aging cohorts, functional dependence, longitudinal study, objective cognition, subjective memory

## Abstract

**INTRODUCTION:**

The prognostic value of concurrent changes in subjective memory and objective cognition for later functional dependence remains unclear across populations.

**METHODS:**

We conducted a harmonized multicohort longitudinal analysis of the China Health and Retirement Longitudinal Study (CHARLS), US Health and Retirement Study (HRS), English Longitudinal Study of Ageing (ELSA), and Survey of Health, Ageing and Retirement in Europe (SHARE). Subjective memory and objective cognition were assessed at baseline (T0) and first follow‐up (T1). Participants were classified into four trajectory groups, and incident functional dependence after T1 was examined using cohort‐specific Cox models and pooled random‐effects meta‐analysis. Sensitivity analyses addressed alternative decline thresholds, continuous change‐score models, fixed follow‐up windows, baseline‐level adjustment, multiple imputation, and competing‐risk models treating death as a competing event in cohorts with adequate mortality information.

**RESULTS:**

Among 86,562 participants included in trajectory derivation and 62,329 included in fully adjusted outcome analyses, concordant decline showed the strongest and most consistent association with incident functional dependence. In pooled Cox analyses, concordant decline was associated with the greatest risk increase (hazard ratio [HR] 1.470, 95% confidence interval [CI] 1.381–1.566), with smaller associations observed for subjective‐only decline (HR 1.222, 95% CI 1.055–1.415) and objective‐only decline (HR 1.116, 95% CI 1.038–1.200). In competing‐risk analyses across CHARLS, HRS, and SHARE, concordant decline remained the strongest association after accounting for death (pooled subdistribution HR 1.386, 95% CI 1.273–1.509).

**DISCUSSION:**

Joint cognitive change, especially concordant decline, may improve identification of older adults at risk of future functional dependence.

## Highlights

- Joint subjective–objective cognitive change predicted functional dependence.
- Concordant decline showed the strongest and most reproducible risk signal.
- Subjective‐only decline retained prognostic value in pooled analyses.
- Combined subjective–objective assessment may improve risk stratification.

## Graphical abstract

Concordant subjective‐memory and objective‐cognition decline showed the strongest and most reproducible association with incident functional dependence across four aging cohorts.

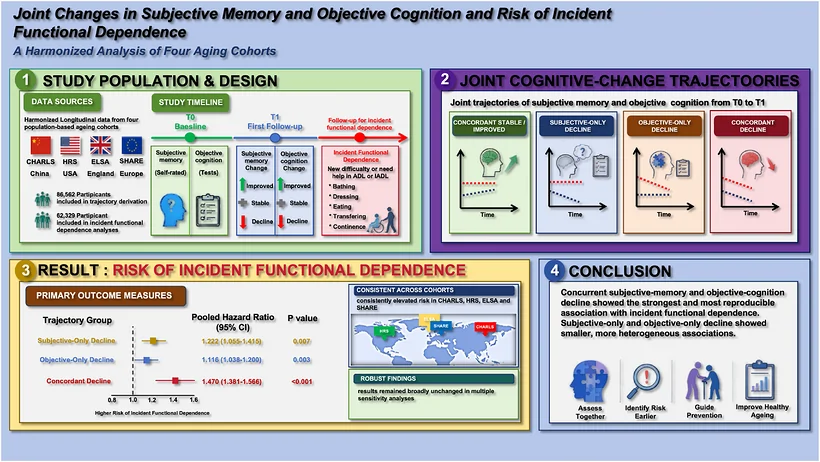

## 1 BACKGROUND

Population aging has made cognitive decline and functional dependence major public health concerns worldwide.^1^, ^2^ Among older adults, functional dependence is particularly significant because it is closely associated with reduced quality of life, increased care requirements, and greater medical and social burden.^3^ Cognitive decline has long been recognized as a major contributor to loss of independence in later life, and longitudinal studies have consistently demonstrated that poorer cognitive performance is associated with subsequent disability and decline in daily functioning.^4^, ^5^

Within the broad domain of cognitive impairment, subjective memory and objective cognition represent two related but distinct dimensions.^6^ Subjective memory reflects an individual's self‐perceived change in memory ability,^7^ whereas objective cognition refers to performance measured using standardized cognitive assessments.^8^ Contemporary frameworks suggest that subjective memory decline may occur even in the absence of measurable impairment on neuropsychological testing and may be influenced not only by early neurodegenerative processes but also by depressive symptoms, health‐related beliefs, and other psychosocial factors.^9^ Accordingly, subjective memory and objective cognitive performance may be either concordant or discordant within individuals.

Both dimensions have significant clinical implications. Subjective memory complaints have been associated with subsequent decline in cognitive performance and may detect early changes that are not yet fully identifiable through formal testing.^10^ In contrast, objective cognitive decline has consistently been linked to later disability and reduced functional independence, underscoring its central role in the development of dependence in old age.^11^, ^12^ However, many prior studies have examined subjective memory and objective cognition separately rather than considering their joint patterns of change.^13^ Previous research suggests that the prognostic value of subjective memory decline may depend on concurrent objective cognitive status, and discordance between subjective and objective cognition may reflect distinct clinical profiles.^14^, ^15^ Therefore, assessing concordance or discordance between subjective memory change and objective cognitive change may yield additional insights for identifying individuals at increased risk of subsequent functional dependence.

Another limitation of the existing literature is that evidence regarding subjective memory and objective cognition has largely been derived from single‐cohort studies, making it difficult to determine whether these associations are reproducible across populations.^16^, ^17^ Harmonized multinational aging datasets provide an important opportunity to address this limitation by enabling comparable analyses across diverse social, cultural, and health‐system contexts. In the present study, we used harmonized longitudinal data from four large aging cohorts to investigate whether joint changes in subjective memory and objective cognition between baseline and the first follow‐up were associated with incident functional dependence. Participants were classified into concordant decline, subjective‐only decline, objective‐only decline, and neither‐decline groups according to whether decline in the two domains occurred jointly or separately over time. By focusing on concordant and discordant change patterns rather than either domain alone, this study addressed key limitations of prior single‐cohort and single‐domain research and evaluated the cross‐cohort robustness and prognostic value of these joint trajectories for subsequent functional dependence.

**RESEARCH IN CONTEXT**

1. **Systematic review**: We reviewed the literature on subjective memory, objective cognition, and later functional dependence in older adults. Prior studies have shown that subjective memory complaints and objective cognitive decline are each associated with adverse functional outcomes, but these domains have usually been examined separately rather than as joint longitudinal change patterns. In addition, most evidence has come from single‐cohort or single‐domain analyses, limiting understanding of whether concordant and discordant subjective–objective cognitive changes are reproducible across populations with different social, cultural, and health‐system contexts.
2. **Interpretation**: Using harmonized longitudinal data from the China Health and Retirement Longitudinal Study; US Health and Retirement Study; English Longitudinal Study of Ageing; and Survey of Health, Ageing and Retirement in Europe, we found that joint changes in subjective memory and objective cognition were differentially associated with incident functional dependence. Concordant decline showed the strongest and most reproducible association across cohorts, whereas subjective‐only decline retained prognostic value and objective‐only decline showed a smaller, more context‐dependent association. These findings were supported by alternative‐threshold analyses, continuous change‐score models, multiple imputation, fixed‐window analyses, baseline‐level adjustment sensitivity analysis, and competing‐risk models. Together, the results suggest that subjective memory change and objective cognitive decline provide complementary rather than competing information for identifying older adults at increased risk of future dependence.
3. **Future directions**: Future studies should validate these findings in additional populations and examine longer term cognitive trajectories using more than two observation points. Further work should also assess whether broader subjective cognitive domains, domain‐specific objective cognitive measures, and more refined definitions of functional dependence improve risk stratification. Integrating harmonized multicohort data with biological, psychosocial, cultural, and health‐system factors may help clarify why concordant decline is more reproducible across populations, whereas discordant patterns show greater cohort‐specific variability.

## 2 METHODS

### 2.1 Data sources, study design, and population

This was a harmonized multicohort longitudinal study based on four population‐based aging surveys: the China Health and Retirement Longitudinal Study (CHARLS); the US Health and Retirement Study (HRS); the English Longitudinal Study of Ageing (ELSA); and the Survey of Health, Ageing and Retirement in Europe (SHARE). CHARLS initiated its national baseline survey in 2011 to 2012, HRS has been conducted biennially since 1992, ELSA began in 2002 with biennial follow‐up, and SHARE commenced panel data collection in 2004. To enhance cross‐national comparability, harmonized data products and cohort‐aligned variable definitions were used whenever possible.

For the present analysis, we used harmonized longitudinal observations from the four cohorts and defined two individual‐level analytic time points, T0 and T1. For each participant, all eligible visits were ordered chronologically, and the first and second visits with valid data on both subjective memory and objective cognition were defined as T0 and T1, respectively. Changes between T0 and T1 were used to derive the trajectory groups, whereas follow‐up visits after T1 were used to ascertain incident functional dependence. Participants were included in the trajectory derivation analyses if they had non‐missing subjective memory and objective cognition measures at both T0 and T1. For the time‐to‐event analyses, we further excluded participants with functional dependence already present at either T0 or T1, so that only incident cases occurring after T1 were counted as outcomes. We additionally required at least one follow‐up assessment after T1 for inclusion in the Cox regression analyses.

### 2.2 Harmonization of subjective memory and objective cognition

Subjective memory and objective cognition were harmonized across the four cohorts to capture two related but distinct dimensions of cognitive aging. Subjective memory data were obtained from cohort‐specific self‐assessment questions administered at T0 and T1. Responses were recoded such that higher values indicated poorer perceived memory abilities and then standardized within each cohort to generate harmonized subjective memory *z* scores. Objective cognitive data were derived from cohort‐specific cognitive test batteries, which were first adjusted to produce comparable scores within each cohort before being standardized to generate objective cognitive *z* scores. This approach was consistent with the conceptual distinction between self‐perceived cognitive decline and objectively measured cognitive performance, as emphasized in previous research on subjective cognitive aging.

Changes between T0 and T1 were calculated as:

$$dSM=SM_{T1}-SM_{T0}$$

and

$$dOC=OC_{T1}-OC_{T0}$$

A higher value of *dSM* indicated worsening subjective memory, whereas a lower value of *dOC* indicated worsening objective cognition. Details of cohort‐specific source variables and harmonization procedures are provided in Table S1 in supporting information.

### 2.3 Definition of trajectory groups

The primary exposure was the joint pattern of change in subjective memory and objective cognition between T0 and T1. In the primary analysis, decline was defined using a cohort‐specific distribution‐based threshold of 0.5 standard deviations (SDs) of the corresponding change score. Specifically, subjective memory decline was defined as *dSM* ≥ 0.5 × SD(*dSM*), because higher *dSM* values indicated worsening perceived memory. Objective cognitive decline was defined as *dOC* ≤ −0.5 × SD(*dOC*), because lower *dOC* values indicated worsening cognitive performance. The 0.5‐SD threshold was selected a priori as a pragmatic, cohort‐specific distribution‐based threshold for moderate change rather than as a clinically validated diagnostic cut‐off. Half‐SD thresholds have been widely used to aid interpretation of change in health‐outcome measures,^18^ and prior cognitive‐aging research has used a 0.5‐SD decrease from baseline cognitive scores as an interpretive threshold when contextualizing clinically meaningful cognitive decline.^19^ In the present harmonized analysis, the cohort‐specific 0.5‐SD threshold provided a common within‐cohort metric across cohorts using different subjective‐memory questions and cognitive test batteries, while reducing classification based on small fluctuations and retaining adequate sample sizes across trajectory groups.

Based on the joint presence or absence of subjective memory decline and objective cognitive decline, participants were classified into four mutually exclusive groups: (1) concordant stable or improved, (2) subjective‐only decline, (3) objective‐only decline, and (4) concordant decline. The concordant stable or improved group served as the reference category in all regression models. This framework was designed to distinguish concordant from discordant patterns of change and to evaluate whether self‐perceived memory worsening and objectively measured cognitive decline carried different prognostic implications when they occurred together or in isolation.

### 2.4 Covariate selection

The fully adjusted models included baseline age, sex, education, marital status, self‐rated health, depressive symptoms, smoking status, current drinking, baseline subjective memory, and baseline objective cognition. These covariates were selected a priori because they are plausibly associated with both cognitive change and later functional dependence, and because subjective memory complaints may be influenced by affective and health‐related perceptions in addition to neurocognitive processes. Education and marital status were harmonized into comparable cohort‐specific categorical or binary variables. Self‐rated health and depressive symptom burden were standardized within each cohort to enhance comparability across different survey instruments. Smoking history was harmonized as ever smoking, and alcohol use as current drinking. Baseline subjective memory and baseline objective cognition were included to account for initial levels of self‐perceived memory and cognitive performance, thereby reducing confounding by baseline status.

### 2.5 Outcome definition

The primary outcome was incident functional dependence after T1. Functional dependence was harmonized using cohort‐specific information on activities of daily living (ADL) and instrumental activities of daily living (IADL), following the conceptual frameworks established by Katz and by Lawton and Brody.^20^ ADL reflects basic self‐care function, whereas IADL reflects more complex tasks required for independent living. In the main analysis, participants were classified as functionally dependent if they had dependence in either ADL or IADL, operationalized as at least one ADL limitation or at least one IADL limitation based on available cohort‐specific ADL/IADL summary measures or harmonized item‐based counts. Thus, the primary harmonized cut‐off was ADL limitation count ≥ 1 or IADL limitation count ≥ 1 in each cohort. Cohort‐specific functional dependence indicators were derived from available ADL/IADL counts or existing dependence variables while preserving conceptual comparability across cohorts. Participants with prevalent functional dependence at either T0 or T1 were excluded so that only incident cases arising after T1 were counted as outcomes. Follow‐up began at T1, and incident functional dependence was defined as the first newly observed functional dependence after T1. Time‐to‐event was calculated from T1 to the first incident event or to censoring at the last available post‐T1 follow‐up. Because the exact operationalization of ADL/IADL measures differed somewhat across cohorts, harmonization enhanced conceptual comparability rather than forced identity of raw‐item definitions. Additional sensitivity analyses using alternative outcome definitions in ELSA and SHARE are presented in Table S2 in supporting information.

### 2.6 Statistical analysis

Baseline characteristics were summarized by trajectory group within each cohort. Continuous variables were presented as mean ± SD and compared using one‐way analysis of variance, whereas categorical variables were presented as number (percentage) and compared using chi‐squared tests. The primary inferential analyses used Cox proportional hazards models to estimate the associations between trajectory group membership and incident functional dependence. We fitted cohort‐specific crude models, age‐ and sex‐adjusted models, and fully adjusted models, with the fully adjusted model prespecified as the primary model for inference. Hazard ratios (HRs) and 95% confidence intervals (CIs) were estimated separately within each cohort using the concordant stable or improved group as the reference. Estimates were pooled using random‐effects meta‐analysis, and between‐cohort heterogeneity was quantified using the *I* ^2^ statistic and the heterogeneity *p* value.

Missing data in the primary analyses were handled using a complete‐case approach at each analytic stage. Participants with missing subjective memory or objective cognition at either T0 or T1 were excluded from trajectory derivation. For the Cox models, participants missing any covariate required for the fully adjusted model were excluded from the corresponding cohort‐specific complete‐case analysis. To clarify the sources of sample reduction, exclusions due to outcome eligibility criteria were distinguished from exclusions due to missing model covariates, and baseline characteristics of included and excluded participants were compared using standardized mean differences. Missingness of variables included in the fully adjusted models among outcome‐eligible participants is summarized in Table S3 in supporting information.

As a missing‐data sensitivity analysis, multiple imputation by chained equations was performed separately within each cohort among participants otherwise eligible for the incident functional‐dependence analysis. Twenty imputed datasets were generated. The imputation models included the trajectory group, all variables in the fully adjusted Cox model, the event indicator, follow‐up time, and the Nelson–Aalen cumulative hazard estimate. Continuous variables were imputed using predictive mean matching, binary variables using logistic regression, and categorical education using multinomial logistic regression. The fully adjusted Cox model was fitted in each imputed dataset, cohort‐specific estimates were combined using Rubin rules, and the resulting estimates were pooled across cohorts using restricted maximum likelihood random‐effects meta‐analysis.

To assess the robustness of the findings to exposure and outcome definitions, we conducted three additional sensitivity analyses. First, alternative trajectory thresholds were examined using more lenient 0.3‐SD and more stringent 0.7‐SD cut‐offs for subjective memory decline and objective cognitive decline. Second, to address the potential information loss caused by dichotomizing continuous change scores, we modeled subjective memory worsening and objective cognition worsening as continuous variables. The two change scores were standardized within each cohort, and the sign of *dOC* was reversed so that higher values consistently indicated greater worsening. Both continuous change scores were entered simultaneously in the fully adjusted Cox model; HRs therefore represented the association with incident functional dependence per 1‐SD increase in worsening, mutually adjusted for change in the other cognitive domain. Third, to examine the robustness of the findings to cohort‐specific differences in functional‐dependence operationalization, we performed additional outcome‐definition sensitivity analyses in ELSA and SHARE: an ADL‐only outcome in ELSA and a stricter definition of ADL ≥ 1 or IADL ≥ 2 in SHARE.

To evaluate whether between‐cohort differences in follow‐up duration influenced the findings, we conducted additional fixed‐window sensitivity analyses. Follow‐up time was administratively truncated at 3 years and 5 years after T1. Events occurring within the specified window were counted as incident functional dependence, events occurring after the window were censored at the window boundary, and participants without an event were censored at the earlier of their last observed follow‐up or the window boundary. The fully adjusted Cox models were refitted within each cohort using the same covariate set as the primary analysis, and cohort‐specific estimates were pooled using restricted maximum likelihood random‐effects meta‐analysis.

Because the trajectory exposure was derived from changes between T0 and T1, and the fully adjusted model included baseline subjective memory and baseline objective cognition, we conducted an additional sensitivity analysis to evaluate the influence of baseline‐level adjustment. Specifically, we refitted the fully adjusted Cox models after removing baseline subjective memory and baseline objective cognition from the covariate set while retaining all other covariates and using the same complete‐case analytic samples. Cohort‐specific estimates were pooled using restricted maximum likelihood random‐effects meta‐analysis.

As an additional sensitivity analysis, death was evaluated as a competing event in cohorts with adequate mortality follow‐up information. The event‐status variable distinguished censoring, incident functional dependence, and death before observed functional dependence, with death before functional dependence treated as the competing event. Fine–Gray subdistribution hazards models were fitted to estimate subdistribution HRs for incident functional dependence. These models used the same covariate set as the fully adjusted Cox models and retained concordant stable or improved as the reference group. The analysis was conducted in CHARLS, HRS, and SHARE; ELSA was not included because the mortality information available in the harmonized analytic data did not fully align with the post‐T1 functional‐dependence follow‐up window. Cohort‐specific Fine–Gray estimates were pooled using restricted maximum likelihood random‐effects meta‐analysis. Separately, the proportional hazards assumption for the primary cohort‐specific fully adjusted Cox models was assessed using Schoenfeld residual–based diagnostics.

## 3 RESULTS

### 3.1 Cohort construction and analytic samples

**FIGURE 1:**
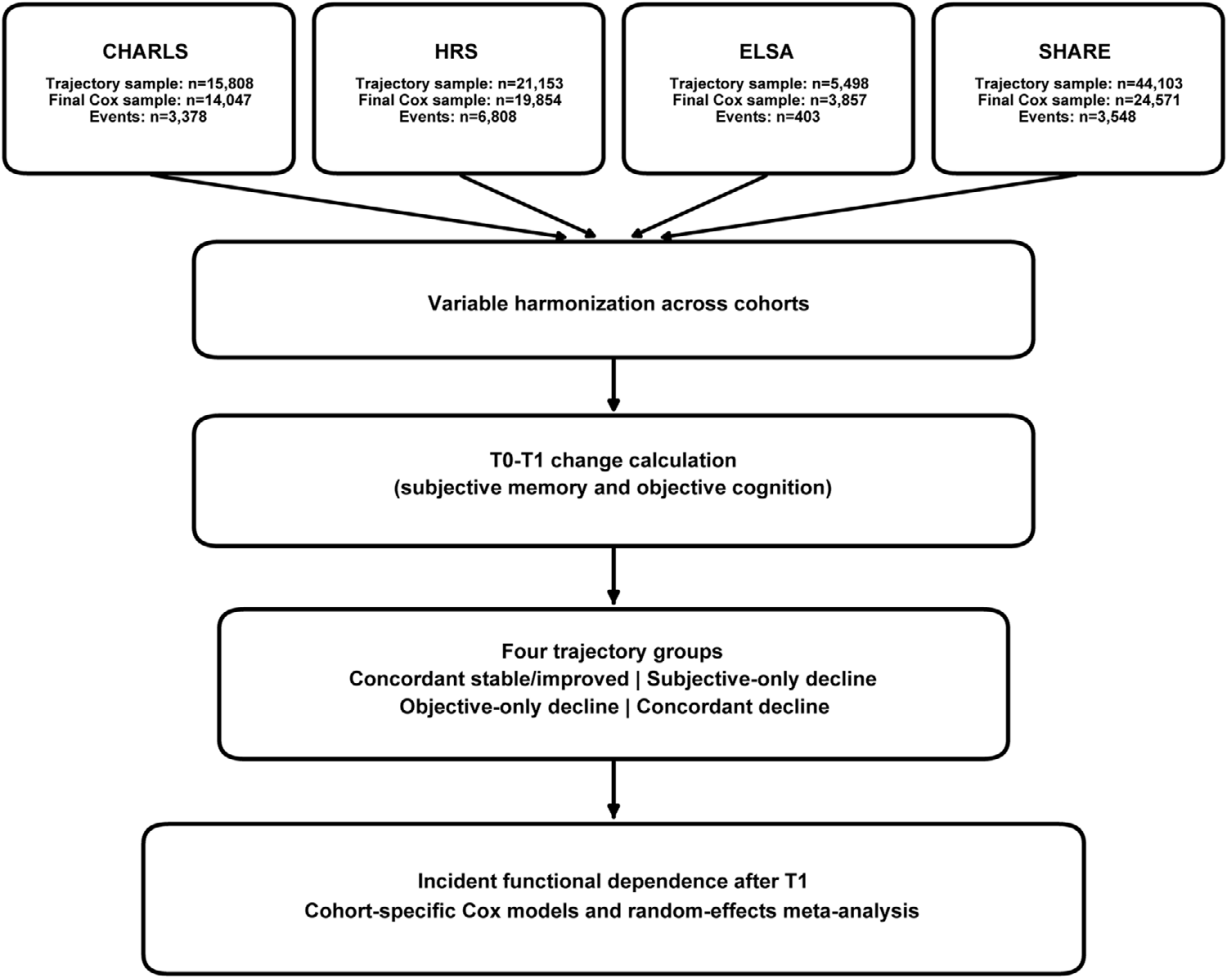
Overall study design and harmonized analytical framework across four cohorts. This figure illustrates the overall study workflow and final analytic samples in CHARLS, HRS, ELSA, and SHARE. Cohort‐specific variables were harmonized, changes in subjective memory and objective cognition between T0 and T1 were calculated, and participants were classified into four trajectory groups. Participants were subsequently followed after T1 for incident functional dependence. Cohort‐specific fully adjusted Cox proportional hazards models were fitted, and the resulting estimates were pooled using random‐effects meta‐analysis. Trajectory sample refers to participants included in trajectory derivation, final Cox sample refers to the complete‐case sample for fully adjusted Cox models, and events refer to incident functional‐dependence events after T1. CHARLS, China Health and Retirement Longitudinal Study; ELSA, English Longitudinal Study of Ageing; HRS, US Health and Retirement Study; SHARE, Survey of Health, Ageing and Retirement in Europe; T0, baseline; T1, first follow‐up.

Figure 1 summarizes the overall study design and harmonized analytical framework across the four cohorts, including variable harmonization, derivation of subjective‐memory and objective‐cognition changes between T0 and T1, classification into four trajectory groups, and subsequent cohort‐specific and pooled risk estimation for incident functional dependence. As shown in Table 1, a total of 15,808 participants from CHARLS; 21,153 from HRS; 5498 from ELSA; and 44,103 from SHARE were included in the trajectory‐derivation analyses. After applying the prespecified outcome eligibility criteria, including the absence of functional dependence at T0 and T1 and the availability of at least one valid post‐T1 outcome assessment, 63,707 participants were eligible for the incident functional‐dependence analysis. Of these, 62,329 were included in the final complete‐case models; thus, only 1378 of 63,707 outcome‐eligible participants (2.2%) were excluded because of missing model covariates. Cohort‐ and variable‐specific missingness among outcome‐eligible participants is summarized in Table S3. The final cohort‐specific Cox analyses included 14,047 participants from CHARLS; 19,854 from HRS; 3857 from ELSA; and 24,571 from SHARE, with 3378, 6808, 403, and 3548 incident functional‐dependence events, respectively. Detailed baseline characteristics by trajectory group within each cohort are provided in Table S4 in supporting information.

**TABLE 1:** Cohort construction and analytic sample characteristics across CHARLS, HRS, ELSA, and SHARE.

Characteristic	CHARLS	HRS	ELSA	SHARE	Summary
Trajectory derivation sample, *n*	15,808	21,153	5498	44,103	86,562
Outcome‐analysis eligible sample before complete‐case restriction, *n*	14,076	19,995	4591	25,045	63,707
Final fully adjusted Cox sample, *n*	14,047	19,854	3857	24,571	62,329
Incident functional dependence events, *n*	3378	6808	403	3548	14,137
Mean follow‐up time, years	4.88	8.71	2.09	4.73	—
Mean age at T0, years	54.95	60.39	65.42	63.84	—
Women, %	50.4	42.7	45.8	42.5	—
Baseline subjective memory *z* score	−0.22	−0.29	−0.16	−0.13	—
Baseline objective cognition *z* score	0.23	0.17	1.09	0.19	—

*Notes*: Outcome‐analysis eligible samples satisfy the post‐T1 outcome and time‐to‐event requirements before complete‐case restriction. Final complete‐case Cox samples additionally contain complete data for all covariates in the fully adjusted model. Descriptive values were recalculated in the final complete‐case Cox samples.

Abbreviations: CHARLS, China Health and Retirement Longitudinal Study; ELSA, English Longitudinal Study of Ageing; HRS, US Health and Retirement Study; SHARE, Survey of Health, Ageing and Retirement in Europe; T0, baseline; T1, first follow‐up.

### 3.2 Definition and distribution of joint trajectory groups

**FIGURE 2:**
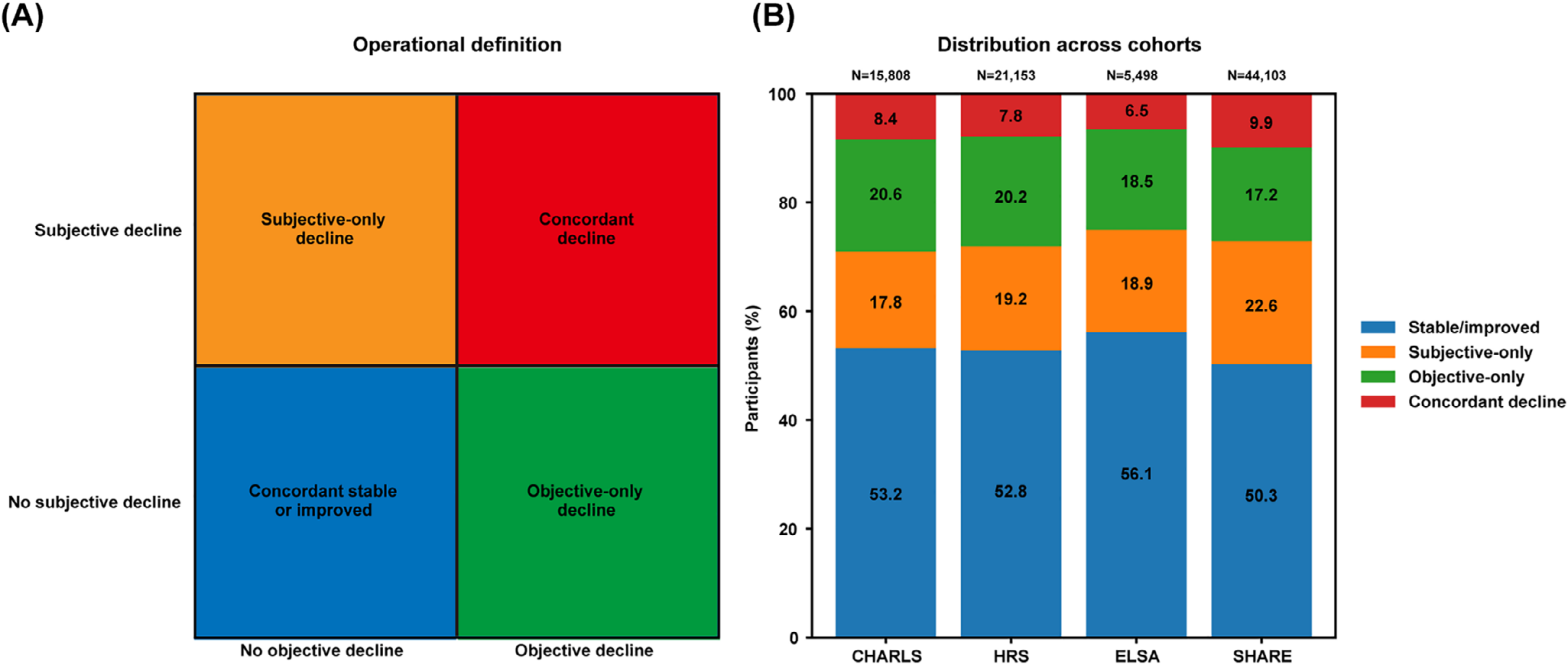
Operational definition and distribution of joint subjective memory–objective cognition trajectory groups. (A) Operational classification of participants according to the presence or absence of subjective memory decline and objective cognitive decline between T0 and T1. (B) Distribution of the four trajectory groups across CHARLS, HRS, ELSA, and SHARE. Numbers inside the stacked bars indicate percentages. Subjective memory decline was defined as dSM ≥ 0.5 × SD(dSM), and objective cognitive decline was defined as dOC ≤ −0.5 × SD(dOC). CHARLS, China Health and Retirement Longitudinal Study; ELSA, English Longitudinal Study of Ageing; HRS, US Health and Retirement Study; SD, standard deviation; SHARE, Survey of Health, Ageing and Retirement in Europe; T0, baseline; T1, first follow‐up.

Figure 2 illustrates both the operational definition and the cross‐cohort distribution of the four joint trajectory groups. Across all cohorts, the concordant stable or improved group was the largest trajectory category, accounting for 53.2% in CHARLS, 52.8% in HRS, 56.1% in ELSA, and 50.3% in SHARE. By contrast, concordant decline consistently represented the smallest subgroup, ranging from 6.5% in ELSA to 9.9% in SHARE. The proportions of subjective‐only decline and objective‐only decline were intermediate and broadly comparable across cohorts, indicating a relatively stable distribution pattern of discordant trajectories across the four datasets. Additional cohort‐specific distribution plots and supplementary descriptive figures are provided in Figure S1 in supporting information.

### 3.3 Crude incidence of functional dependence across trajectory groups

**FIGURE 3:**
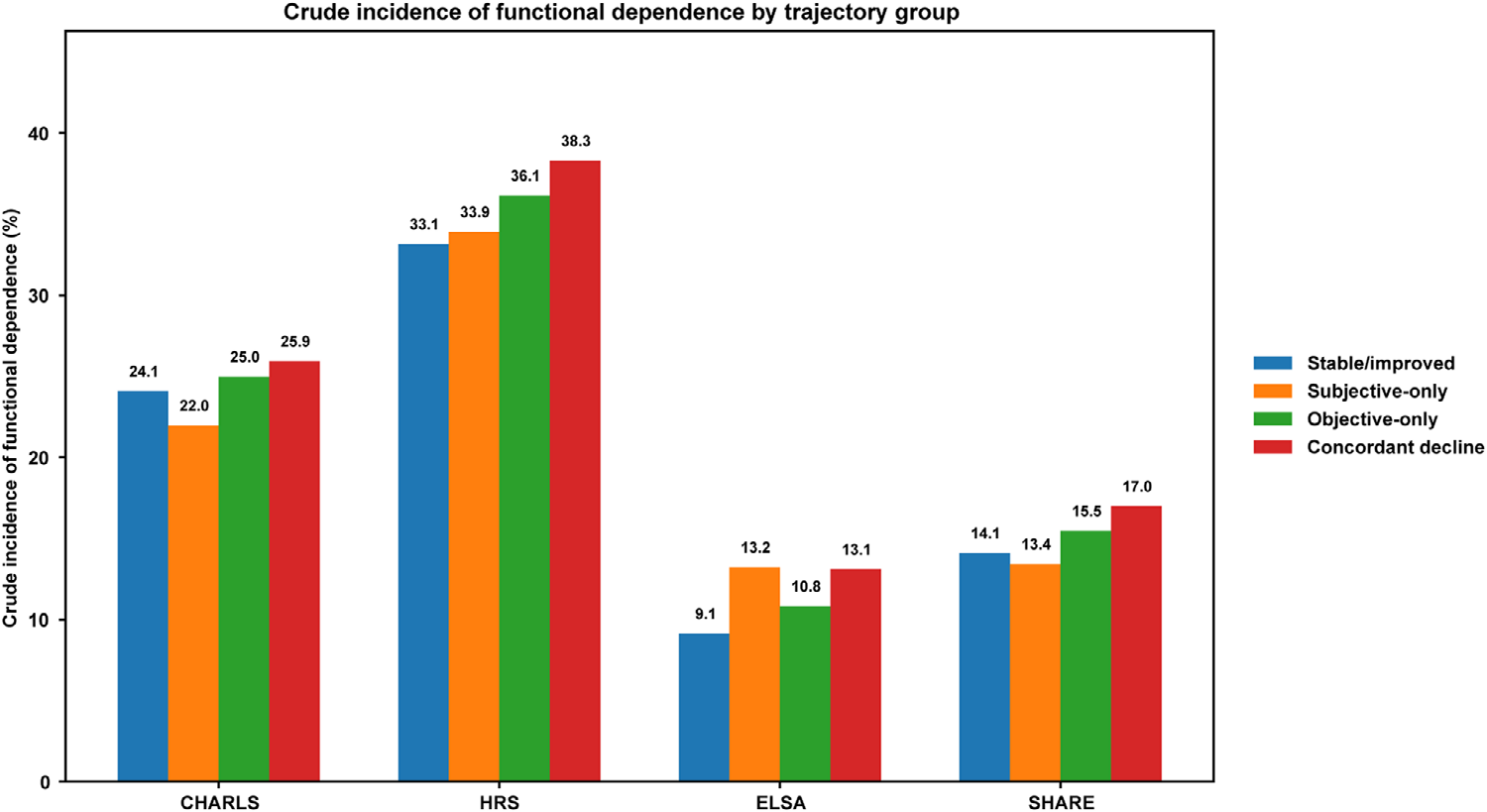
Crude incidence of incident functional dependence across trajectory groups. Bars show the crude percentage of participants who developed incident functional dependence after T1 within each trajectory group and cohort. Numbers above bars indicate crude percentages. Crude incidence was calculated using the final complete‐case Cox model samples. Functional dependence was defined as ADL limitation count ≥ 1 or IADL limitation count ≥ 1 in the primary analysis. ADL, activities of daily living; CHARLS, China Health and Retirement Longitudinal Study; ELSA, English Longitudinal Study of Ageing; HRS, US Health and Retirement Study; IADL, instrumental activities of daily living; SHARE, Survey of Health, Ageing and Retirement in Europe; T0, baseline; T1, first follow‐up used for trajectory derivation.

Crude incidence patterns across the four trajectory groups are presented in Figure 3. In CHARLS, concordant decline showed the highest crude incidence of functional dependence (25.9%), followed by objective‐only decline (25.0%), compared to 24.1% in the concordant stable or improved group. In HRS, concordant decline again showed the highest crude incidence (38.3%), followed by objective‐only decline (36.1%) and subjective‐only decline (33.9%), compared to 33.1% in the concordant stable or improved group. In ELSA, subjective‐only decline showed the highest crude incidence (13.2%), followed closely by concordant decline (13.1%) and objective‐only decline (10.8%), compared to 9.1% in the concordant stable or improved group. In SHARE, concordant decline showed the highest crude incidence (17.0%), followed by objective‐only decline (15.5%), compared to 14.1% in the concordant stable or improved group. These descriptive patterns suggest that concordant decline generally had the highest crude burden of subsequent functional dependence, although ELSA showed a different ordering, with the highest crude incidence observed in the subjective‐only decline group.

### 3.4 Cohort‐specific, fully adjusted associations

The results of the cohort‐specific, fully adjusted Cox models are presented in Table 2. Overall, concordant decline showed the strongest and most consistent association with incident functional dependence and was the only trajectory pattern that was statistically significant in all four cohorts. In CHARLS, subjective‐only decline was associated with a higher risk of functional dependence (HR 1.257, 95% CI 1.125–1.405), as was concordant decline (HR 1.596, 95% CI 1.397–1.824), whereas objective‐only decline was not significantly associated with the outcome (HR 1.043, 95% CI 0.953–1.141). In HRS, all three adverse trajectory patterns were significantly associated with higher risk, with HRs of 1.142 (95% CI 1.066–1.224) for subjective‐only decline, 1.178 (95% CI 1.105–1.255) for objective‐only decline, and 1.464 (95% CI 1.335–1.605) for concordant decline. In ELSA, subjective‐only decline was associated with a higher risk of functional dependence (HR 1.672, 95% CI 1.280–2.183), and concordant decline was also significantly associated with the outcome, although with a slightly smaller estimate and wider confidence interval (HR 1.531, 95% CI 1.033–2.267). The association for objective‐only decline was positive but did not reach statistical significance (HR 1.293, 95% CI 0.985–1.697). Thus, although concordant decline was statistically significant in ELSA in the revised primary analysis, the ELSA pattern remained somewhat distinct because subjective‐only decline showed the largest point estimate. In SHARE, concordant decline was significantly associated with higher risk (HR 1.383, 95% CI 1.229–1.556), whereas subjective‐only decline (HR 1.074, 95% CI 0.978–1.180) and objective‐only decline (HR 1.077, 95% CI 0.982–1.182) were not statistically significant. Taken together, subjective‐only decline was significantly associated with functional dependence in CHARLS, HRS, and ELSA, whereas objective‐only decline was significant only in HRS, indicating greater cross‐cohort variability for the two discordant patterns.

**TABLE 2:** Cohort‐specific fully adjusted associations between joint change patterns in subjective memory and objective cognition and incident functional dependence.

Trajectory group	CHARLS HR (95% CI), *p* value	HRS HR (95% CI), *p* value	ELSA HR (95% CI), *p* value	SHARE HR (95% CI), *p* value
Subjective‐only decline	1.257 (1.125–1.405), *p* < 0.001	1.142 (1.066–1.224), *p* < 0.001	1.672 (1.280–2.183), *p* < 0.001	1.074 (0.978–1.180), *p* = 0.136
Objective‐only decline	1.043 (0.953–1.141), *p* = 0.359	1.178 (1.105–1.255), *p* < 0.001	1.293 (0.985–1.697), *p* = 0.064	1.077 (0.982–1.182), *p* = 0.116
Concordant decline	1.596 (1.397–1.824), *p* < 0.001	1.464 (1.335–1.605), *p* < 0.001	1.531 (1.033–2.267), *p* = 0.034	1.383 (1.229–1.556), *p* < 0.001

***Notes***: The concordant stable or improved group served as the reference category. Cohort‐specific fully adjusted Cox proportional hazards models included baseline age, sex, education, marital status, self‐rated health, depressive symptoms, smoking status, current drinking, baseline subjective memory, and baseline objective cognition. The T0–T1 interval was not included in the finalized models. Complete‐case sample sizes were 14,047 for CHARLS, 19,854 for HRS, 3857 for ELSA, and 24,571 for SHARE; the corresponding numbers of incident functional‐dependence events were 3378, 6808, 403, and 3548, respectively.

Abbreviations: CHARLS, China Health and Retirement Longitudinal Study; CI, confidence interval; ELSA, English Longitudinal Study of Ageing; HR, hazard ratio; HRS, US Health and Retirement Study; SHARE, Survey of Health, Ageing and Retirement in Europe; T0, baseline; T1, first follow‐up.

Crude and age‐ and sex‐adjusted associations, recalculated using the same final complete‐case samples as the fully adjusted models, are presented in Tables S5 and S6 in supporting information, respectively. Cohort‐specific Kaplan–Meier curves showed overall unadjusted differences in the probability of remaining free of functional dependence across trajectory groups in CHARLS (log‐rank *p* = 0.008), HRS (*p* < 0.001), ELSA (*p* = 0.007), and SHARE (*p* < 0.001), as shown in Figure S2A–D in supporting information. Schoenfeld residual–based diagnostics are summarized in Table S7 in supporting information. Comparisons of participants included in and excluded from the final analyses are presented in Table S8 in supporting information. No evidence of non‐proportionality was observed for the primary trajectory terms in CHARLS, ELSA, or SHARE. In HRS, however, evidence of non‐proportionality was observed for objective‐only decline (*p* < 0.001) and concordant decline (*p* = 0.006), whereas the assumption was satisfied for subjective‐only decline (*p* = 0.670).

### 3.5 Pooled meta‐analysis

**FIGURE 4:**
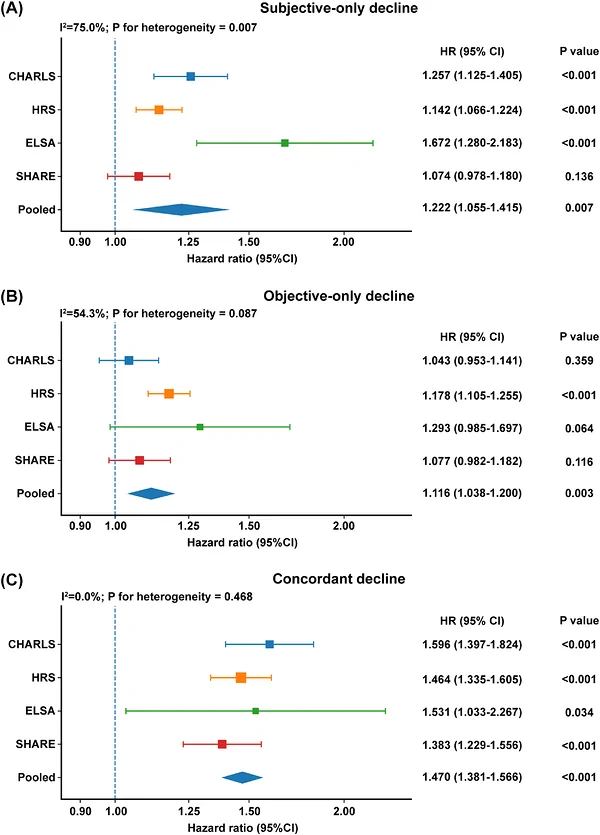
Cohort‐specific and pooled associations between joint change patterns in subjective memory and objective cognition and incident functional dependence. Forest plots show fully adjusted cohort‐specific hazard ratios and pooled random‐effects estimates for (A) subjective‐only decline, (B) objective‐only decline, and (C) concordant decline, compared to the concordant stable or improved group. Right‐hand columns provide HRs with 95% CIs and *p* values. Cohort‐specific models were adjusted for baseline age, sex, education, marital status, self‐rated health, depressive symptoms, smoking status, current drinking, baseline subjective memory, and baseline objective cognition. Pooled estimates were calculated using restricted maximum likelihood random‐effects meta‐analysis. Diamonds indicate pooled estimates. CHARLS, China Health and Retirement Longitudinal Study; CI, confidence interval; ELSA, English Longitudinal Study of Ageing; HR, hazard ratio; HRS, US Health and Retirement Study; SHARE, Survey of Health, Ageing and Retirement in Europe.

The pooled random‐effects meta‐analysis is summarized in Table 3, and the corresponding cohort‐specific and pooled estimates are displayed in Figure 4. Concordant decline showed the strongest pooled association with incident functional dependence (pooled HR 1.470, 95% CI 1.381–1.566), with no measurable between‐cohort heterogeneity (*I* ^2^ = 0.0%). Subjective‐only decline was also associated with a higher risk of functional dependence (pooled HR 1.222, 95% CI 1.055–1.415), although substantial between‐cohort heterogeneity was observed (*I* ^2^ = 75.0%). Objective‐only decline showed a smaller but statistically significant pooled association (pooled HR 1.116, 95% CI 1.038–1.200), with moderate heterogeneity (*I* ^2^ = 54.3%). Overall, the pooled findings demonstrated a graded pattern of association, with concordant decline conferring the greatest and most consistent risk across cohorts, whereas the two discordant patterns were associated with more modest effects and greater cross‐cohort variability.

**TABLE 3:** Random‐effects meta‐analysis of cohort‐specific associations between joint change patterns in subjective memory and objective cognition and incident functional dependence.

Trajectory group	Pooled HR (95% CI)	*p* value	*I* ^2^	*p* value for heterogeneity
Subjective‐only decline	1.222 (1.055–1.415)	0.007	75.0%	0.007
Objective‐only decline	1.116 (1.038–1.200)	0.003	54.3%	0.087
Concordant decline	1.470 (1.381–1.566)	<0.001	0.0%	0.468

***Notes***: Cohort‐specific fully adjusted hazard ratios were pooled using restricted maximum likelihood random‐effects meta‐analysis. The concordant stable or improved group served as the reference category. Between‐cohort heterogeneity was quantified using the *I* ^2^ statistic and Cochran *Q* test.

Abbreviations: CI, confidence interval; HR, hazard ratio.

### 3.6 Sensitivity analyses

The overall pattern of association was preserved when alternative thresholds were used to define decline. At the more lenient 0.3‐SD threshold, the pooled hazard ratios were 1.197 (95% CI 1.058–1.355) for subjective‐only decline, 1.100 (95% CI 1.027–1.178) for objective‐only decline, and 1.426 (95% CI 1.345–1.511) for concordant decline. At the more stringent 0.7‐SD threshold, the corresponding pooled HRs were 1.214 (95% CI 1.083–1.360), 1.147 (95% CI 1.062–1.239), and 1.504 (95% CI 1.405–1.610), respectively (Table 4). Concordant decline therefore remained the strongest association under both alternative definitions.

**TABLE 4:** Sensitivity analyses of the associations between joint changes in subjective memory and objective cognition and incident functional dependence.

Threshold	Trajectory group	CHARLS	HRS	ELSA	SHARE	Pooled HR (95% CI)	*p* value	*I* ^2^	*p* value for heterogeneity
**Panel A. Alternative thresholds for defining subjective‐memory and objective‐cognition decline**									
0.3 SD	Subjective‐only decline	1.244 (1.107–1.398)	1.142 (1.060–1.230)	1.621 (1.225–2.146)	1.067 (0.965–1.178)	1.197 (1.058–1.355)	0.004	69.1%	0.021
	Objective‐only decline	1.026 (0.942–1.117)	1.164 (1.097–1.235)	1.145 (0.885–1.481)	1.087 (0.997–1.184)	1.100 (1.027–1.178)	0.006	50.2%	0.110
	Concordant decline	1.541 (1.359–1.748)	1.411 (1.297–1.534)	1.565 (1.104–2.220)	1.355 (1.213–1.513)	1.426 (1.345–1.511)	<0.001	0.0%	0.452
0.7 SD	Subjective‐only decline	1.275 (1.145–1.420)	1.155 (1.080–1.235)	1.586 (1.220–2.060)	1.091 (0.997–1.195)	1.214 (1.083–1.360)	<0.001	70.1%	0.018
	Objective‐only decline	1.074 (0.977–1.181)	1.217 (1.138–1.303)	1.328 (0.986–1.789)	1.100 (0.993–1.218)	1.147 (1.062–1.239)	<0.001	52.4%	0.098
	Concordant decline	1.643 (1.426–1.894)	1.482 (1.341–1.638)	1.731 (1.151–2.601)	1.414 (1.243–1.609)	1.504 (1.405–1.610)	<0.001	0.0%	0.405

Continuous exposure	CHARLS	HRS	ELSA	SHARE	Pooled HR (95% CI)	*p* value	*I* ^2^	*p* for heterogeneity
**Panel B. Continuous change‐score analyses**								
Subjective‐memory worsening, per 1‐SD increase	1.153 (1.101–1.207)	1.054 (1.025–1.084)	1.240 (1.106–1.390)	1.060 (1.016–1.105)	1.110 (1.040–1.184)	0.002	82.2%	<0.001
Objective‐cognition worsening, per 1‐SD increase	1.104 (1.065–1.145)	1.136 (1.107–1.167)	1.099 (0.998–1.209)	1.112 (1.072–1.153)	1.121 (1.100–1.142)	<0.001	0.0%	0.566

*Notes*: Values are HRs and 95% CIs unless otherwise indicated. The concordant stable or improved group was the reference category in the alternative‐threshold analyses. All cohort‐specific models were adjusted for baseline age, sex, education, marital status, self‐rated health, depressive symptoms, smoking status, current drinking, baseline subjective memory, and baseline objective cognition. The T0–T1 interval was not included in these models. For continuous analyses, subjective‐memory worsening and objective‐cognition worsening were standardized within each cohort and entered simultaneously in the same model. The sign of objective cognitive change was reversed so that higher values indicated greater worsening. Pooled estimates were calculated using REML random‐effects meta‐analysis. The primary 0.5‐SD analysis is reported in Tables 2 and 3 and is not repeated in this sensitivity‐analysis table.

Abbreviations: CHARLS, China Health and Retirement Longitudinal Study; CI, confidence interval; ELSA, English Longitudinal Study of Ageing; HR, hazard ratio; HRS, US Health and Retirement Study; REML, restricted maximum likelihood; SD, standard deviation; SHARE, Survey of Health, Ageing and Retirement in Europe; T0, baseline; T1, first follow‐up.

The continuous change‐score analyses yielded findings consistent with the primary categorical analysis (Table 4). Each 1‐SD increase in subjective‐memory worsening was associated with a higher risk of incident functional dependence (pooled HR 1.110, 95% CI 1.040–1.184), although between‐cohort heterogeneity was substantial (*I* ^2^ = 82.2%). Each 1‐SD increase in objective‐cognition worsening was also associated with higher risk (pooled HR 1.121, 95% CI 1.100–1.142), with no measurable between‐cohort heterogeneity (*I* ^2^ = 0.0%). These findings indicate that the main results were not solely attributable to dichotomization of the change scores or to the specific 0.5‐SD threshold.

Multiple‐imputation analyses among all 63,707 outcome‐eligible participants produced results that were materially consistent with the complete‐case analyses (Table S9 in supporting information). The pooled HRs were 1.223 (95% CI 1.050–1.425; *p* = 0.010) for subjective‐only decline, 1.118 (95% CI 1.043–1.199; *p* = 0.002) for objective‐only decline, and 1.465 (95% CI 1.376–1.559; *p* < 0.001) for concordant decline. Concordant decline therefore remained the strongest and most consistent association. The direction of association was preserved in all cohort‐specific analyses, although the ELSA estimate for concordant decline was attenuated and was no longer statistically significant after multiple imputation.

To address death as a competing event, Fine–Gray subdistribution hazards models were fitted in CHARLS, HRS, and SHARE (Table S10 in supporting information). The final competing‐risk model samples included 14,296 participants in CHARLS; 20,435 in HRS; and 25,485 in SHARE, with 3378, 6808, and 3559 functional‐dependence events and 599, 3469, and 831 competing deaths, respectively. The analysis was conducted in CHARLS, HRS, and SHARE; ELSA was not included because the mortality information available in the harmonized analytic data did not fully align with the post‐T1 functional‐dependence follow‐up window. In cohort‐specific Fine–Gray analyses, concordant decline remained positively associated with incident functional dependence in all three cohorts, with subdistribution HRs of 1.528 (95% CI 1.341–1.740) in CHARLS, 1.367 (95% CI 1.243–1.504) in HRS, and 1.294 (95% CI 1.151–1.455) in SHARE. In random‐effects meta‐analysis, the pooled subdistribution HRs were 1.160 (95% CI 1.065–1.265; *p* < 0.001) for subjective‐only decline, 1.071 (95% CI 1.010–1.136; *p* = 0.023) for objective‐only decline, and 1.386 (95% CI 1.273–1.509; *p* < 0.001) for concordant decline. Thus, accounting for death modestly attenuated the estimates relative to the primary Cox models but did not alter the overall pattern of findings.

Because follow‐up duration differed across cohorts, we performed additional fixed‐window sensitivity analyses (Table S11 in supporting information). In the complete‐case samples, median follow‐up ranged from 2.08 years in ELSA to 7.83 years in HRS. When follow‐up was administratively truncated at 3 years after T1, concordant decline remained the strongest association with incident functional dependence (pooled HR 1.495, 95% CI 1.343–1.665; *p* < 0.001). Subjective‐only decline also remained associated with higher risk (pooled HR 1.241, 95% CI 1.046–1.472; *p* = 0.013), whereas objective‐only decline showed a positive but non‐significant association (pooled HR 1.125, 95% CI 0.994–1.273; *p* = 0.062). In the 5‐year restricted analysis, the corresponding pooled HRs were 1.209 (95% CI 1.029–1.421; *p* = 0.021), 1.161 (95% CI 1.037–1.299; *p* = 0.009), and 1.504 (95% CI 1.376–1.643; *p* < 0.001), respectively. These findings suggested that the main pattern, particularly the predominance of concordant decline, was not driven solely by unequal follow‐up duration across cohorts.

We also examined whether adjustment for baseline subjective memory and baseline objective cognition influenced the estimates (Table S12 in supporting information). When baseline subjective memory and baseline objective cognition were removed from the fully adjusted models while retaining the same complete‐case samples and all other covariates, the pooled estimates were attenuated. The pooled HRs were 1.116 (95% CI 0.975–1.276; *p* = 0.110) for subjective‐only decline, 1.030 (95% CI 0.952–1.114; *p* = 0.466) for objective‐only decline, and 1.262 (95% CI 1.180–1.350; *p* < 0.001) for concordant decline. Thus, concordant decline remained significantly associated with incident functional dependence even without adjustment for baseline subjective memory and baseline objective cognition, whereas the two discordant patterns were more sensitive to baseline‐level adjustment.

## 4 DISCUSSION

In this harmonized multicohort longitudinal study, joint changes in subjective memory and objective cognition were differentially associated with subsequent functional dependence. Concordant decline showed the strongest and most consistent prognostic association across the cohort‐specific analyses and the pooled meta‐analysis, with a pooled HR of 1.470 (95% CI 1.381–1.566) and no measurable between‐cohort heterogeneity. Subjective‐only decline was also associated with incident functional dependence at the pooled level (HR 1.222, 95% CI 1.055–1.415), although the magnitude of the association varied substantially across cohorts. Objective‐only decline showed a smaller but statistically significant pooled association (HR 1.116, 95% CI 1.038–1.200), with greater cross‐cohort variability than concordant decline. These patterns were preserved across alternative decline thresholds, continuous change‐score models, fixed follow‐up windows, baseline‐level adjustment sensitivity analysis, multiple imputation, and competing‐risk analysis, supporting the robustness of the main findings.

These findings support the view that subjective memory and objective cognition are related but non‐interchangeable indicators of cognitive aging.^13^, ^17^ Their joint change pattern appeared to provide prognostic information beyond either domain alone, consistent with frameworks suggesting that subjective cognitive decline may emerge before or alongside measurable impairment.^21^ By examining coordinated changes across four cohorts, our study extends prior work that has often considered subjective complaints and objective cognition separately.^15^

The predominance of concordant decline is the central message of the present study. Conceptually, subjective memory worsening may reflect self‐awareness of change, whereas objective decline reflects measurable deterioration on standardized testing; when these two processes co‐occur, they may indicate a more functionally salient stage of cognitive deterioration.^21^ This interpretation is clinically plausible because previous longitudinal work has shown that poorer cognitive performance is associated with later disability and functional decline, particularly in domains relevant to independent living.^22^ Our results build on that literature by showing that the highest risk was not defined by objective decline alone, but by the convergence of subjective and objective worsening over time. The fact that concordant decline remained the strongest predictor across the primary and sensitivity analyses further suggests that it represents a reproducible high‐risk phenotype rather than a threshold‐dependent artifact.

Several sensitivity analyses qualified but did not materially change this interpretation. Fixed‐window analyses showed that the predominance of concordant decline was preserved after administrative truncation at 3 and 5 years, suggesting that the primary findings were not simply driven by longer follow‐up in some cohorts. When baseline subjective memory and baseline objective cognition were removed from the model, the associations were attenuated, particularly for the two discordant patterns, but concordant decline remained significantly associated with incident functional dependence. This pattern is consistent with the rationale for retaining baseline levels in the primary model, while indicating that the concordant‐decline finding was not entirely dependent on baseline‐level adjustment. Finally, when death was treated as a competing event in CHARLS, HRS, and SHARE, the estimates were modestly attenuated, but concordant decline remained the strongest association with incident functional dependence. Thus, although standard Cox models may somewhat overestimate cumulative incidence–related associations in older populations, the concordant‐decline association was not explained solely by unequal follow‐up, baseline‐level adjustment, or informative censoring due to death.

A second notable finding is the prognostic relevance of subjective‐only decline. In cohort‐specific analyses, subjective‐only decline was significantly associated with incident functional dependence in CHARLS, HRS, and ELSA, and it remained significant in the pooled meta‐analysis despite substantial between‐cohort heterogeneity. This suggests that worsening self‐perceived memory should not be dismissed simply because objective decline is not yet clearly detectable. Current frameworks of subjective cognitive decline emphasize that self‐perceived worsening may precede the detection threshold of conventional cognitive tests, and longitudinal evidence indicates that increasing subjective memory complaints may predict later decline in memory, IADL, and social participation.^10^, ^21^ The prominence of subjective‐only decline in ELSA, in which it showed the largest point estimate and remained more robust after multiple imputation, further illustrates this heterogeneity. This may reflect ELSA's smaller sample; fewer events; shorter post‐T1 follow‐up; and differences in cognitive testing, subjective‐memory wording, and ADL/IADL operationalization. Together, these findings suggest that subjective‐only decline may capture early vulnerability, mood‐related perception, health expectations, or reduced reserve not fully reflected in objective cognitive scores.^16^

By contrast, objective‐only decline showed a weaker and less consistent association with later functional dependence than concordant decline. In the pooled analysis, objective‐only decline was associated with a modest but statistically significant increase in risk (HR 1.116, 95% CI 1.038–1.200), although the cohort‐specific association reached statistical significance only in HRS. One possible explanation is methodological: harmonized multicohort analyses necessarily prioritize cross‐survey comparability, which may reduce sensitivity to domain‐specific cognitive features that are particularly relevant to everyday functioning. A second explanation is conceptual: discordance between subjective and objective cognition may itself be informative rather than simply reflecting measurement error, because discordant groups may differ in depressive symptom burden, awareness of decline, health expectations, and other contextual influences on self‐report.^15^, ^16^ Our findings are consistent with this interpretation and suggest that objective cognitive decline in the absence of concurrent self‐perceived worsening carries a modest and context‐dependent risk signal but is less consistently associated with subsequent loss of independence than concordant decline.

The between‐cohort heterogeneity observed in the pooled analyses was also informative. Heterogeneity was substantial for subjective‐only decline and moderate for objective‐only decline, whereas no measurable between‐cohort heterogeneity was observed for concordant decline. This gradient is plausible because subjective evaluations of memory are influenced not only by neurocognitive change itself but also by depressive and anxiety symptoms, self‐perceptions, and broader contextual factors that may shape how cognitive problems are experienced and reported.^9^, ^16^ In addition, cross‐cultural studies suggest that the associations between subjective cognitive complaints, mood, and objective cognition may vary across populations, supporting the possibility that reporting norms and sociocultural context contribute to between‐cohort heterogeneity.^23^, ^24^ From this perspective, the consistency of the association for concordant decline across CHARLS, HRS, ELSA, and SHARE strengthens the interpretation that concurrent subjective and objective worsening represents a comparatively transportable and clinically meaningful risk pattern across populations.

These findings have several implications for research and practice. They support a more integrated framework for cognitive risk stratification in aging populations, in which subjective memory change and objective cognitive decline are interpreted as complementary rather than competing indicators.^13^, ^21^ They also suggest that repeated assessment may be more informative than single time‐point classification, because the prognostic signal in our study was defined by change between T0 and T1 rather than by a single baseline measure—an interpretation consistent with prior longitudinal work on the temporal interplay between subjective memory complaints and objective cognitive change.^17^ Clinically, individuals with concordant decline may represent the subgroup at greatest risk for later dependence and therefore may warrant closer surveillance, whereas subjective‐only decline may identify an earlier but still meaningful stage of vulnerability.^10^ More broadly, our study illustrates the value of harmonized multinational aging datasets for testing whether cognitive risk patterns are reproducible across diverse social, cultural, and health‐system settings.

Several limitations should be acknowledged. First, harmonization was conceptual rather than item‐identical across cohorts, and residual measurement heterogeneity is unavoidable. This applied to both subjective‐memory assessment and ADL/IADL‐based functional‐dependence definitions, because source questions, response options, and item availability differed across cohorts. Subjective memory was based on self‐report, focused on perceived memory rather than broader subjective cognition, and may have been influenced by depressive symptoms, health perceptions, language, or sociocultural response styles. For transparency, detailed cohort‐specific functional‐dependence operationalization and original subjective‐memory item wording are provided in Tables S13 and S14 in supporting information, respectively. Second, change was defined using two observations rather than longer latent trajectories, and dichotomizing change scores may have reduced information; however, alternative thresholds and continuous change‐score analyses produced broadly consistent findings. Third, although only 2.2% of outcome‐eligible participants were excluded because of missing model covariates and multiple‐imputation analyses were materially consistent, complete‐case analysis may still have introduced selection bias if missingness was related to frailty, cognition, or impending dependence. Fourth, follow‐up duration and outcome ascertainment differed across cohorts. Fixed‐window analyses reduced this concern, but unequal observation structures may still have affected precision, particularly in ELSA, which had the shortest post‐T1 follow‐up. Fifth, competing‐risk analyses for death were limited to CHARLS, HRS, and SHARE because ELSA mortality information did not adequately cover the analytic follow‐up period. Finally, because this was an observational study, the findings should be interpreted as prognostic rather than causal.

## CONFLICT OF INTEREST STATEMENT

The authors declare no conflicts of interest. Author disclosures are available in the Supporting Information.

## CONSENT STATEMENT

All participants provided informed consent in the original CHARLS, HRS, ELSA, and SHARE studies. This secondary analysis used de‐identified publicly available/harmonized data and did not involve direct participant contact.

## Supporting information



This figure shows the cohort‐specific distributions of change in subjective memory and objective cognition between T0 and T1 in CHARLS, HRS, ELSA, and SHARE. Subjective memory change was calculated as *dSM* = *SM*_T1 − *SM*_T0, with higher values indicating worsening subjective memory. Objective cognition change was calculated as *dOC* = *OC*_T1 − *OC*_T0, with lower values indicating worsening objective cognition. Dashed vertical lines indicate the primary cohort‐specific 0.5‐SD decline thresholds. Subjective memory decline was defined as *dSM* ≥ 0.5 × SD(*dSM*), and objective cognitive decline was defined as *dOC* ≤ −0.5 × SD(*dOC*). CHARLS, China Health and Retirement Longitudinal Study; HRS, Health and Retirement Study; ELSA, English Longitudinal Study of Ageing; SHARE, Survey of Health, Ageing and Retirement in Europe; T0, baseline; T1, first follow‐up used for trajectory derivation.

Kaplan–Meier curves show the probability of remaining free of functional dependence after T1 across the four trajectory groups in **(A)** CHARLS, **(B)** HRS, **(C)** ELSA, and **(D)** SHARE. Between‐group differences were evaluated using log‐rank tests. All curves were generated using the same final complete‐case samples as the fully adjusted cohort‐specific Cox models.

Supporting Information

Supporting Information

Supporting Information

Supporting Information

Supporting Information

Supporting Information

Supporting Information

Supporting Information

Supporting Information

Supporting Information

Supporting Information

Supporting Information

Supporting Information

Supporting Information

Supporting Information
