# Supplementary material for: Joint changes in subjective memory and objective cognition and risk of incident functional dependence: A harmonized analysis of four aging cohorts
Source: Alzheimers Dement. 2026 Aug 22;22(8):e71752. doi: 10.1002/alz.71752 (PMC13498894; doi:10.1002/alz.71752)
Supplement: Supplementary file 2 [file ALZ-22-e71752-s002.docx]

**Table S11. Sensitivity analyses using fixed follow-up windows to address between-cohort follow-up heterogeneity**

| **Panel A. Follow-up duration and event distribution in the complete-case analysis sample** | | | | | | | | | |
| --- | --- | --- | --- | --- | --- | --- | --- | --- | --- |
| **Cohort** | **N** | **Events** | **Censored** | **Person-years** | **Events per 100 PY** | **Median follow-up (IQR), years** | **Follow-up range, years** | **Events ≤3 years** | **Events ≤5 years** |
| CHARLS | 14,047 | 3,378 | 10,669 | 68,577.3 | 4.93 | 5.00 (2.08–7.00) | 1.67–7.25 | 1,489 | 2,452 |
| HRS | 19,854 | 6,808 | 13,046 | 172,977.8 | 3.94 | 7.83 (4.50–12.42) | 0.83–18.92 | 1,435 | 2,708 |
| ELSA | 3,857 | 403 | 3,454 | 8,063.2 | 5.00 | 2.08 (2.00–2.17) | 1.17–3.00 | 403 | 403 |
| SHARE | 24,571 | 3,548 | 21,023 | 116,336.2 | 3.05 | 6.33 (2.17–6.67) | 1.25–7.08 | 1,739 | 2,303 |

| **Panel B. Cohort-specific fully adjusted Cox models after administrative truncation at 3 and 5 years** | | | | | | | |
| --- | --- | --- | --- | --- | --- | --- | --- |
| **Window** | **Cohort** | **Trajectory group** | **HR (95% CI)** | **P value** | **N** | **Events** | **Censored** |
| 3-year | CHARLS | Subjective-only decline | 1.304 (1.099–1.547) | 0.002 | 14,047 | 1,489 | 12,558 |
| 3-year | CHARLS | Objective-only decline | 0.997 (0.872–1.140) | 0.965 | 14,047 | 1,489 | 12,558 |
| 3-year | CHARLS | Concordant decline | 1.525 (1.241–1.874) | <0.001 | 14,047 | 1,489 | 12,558 |
| 3-year | HRS | Subjective-only decline | 1.099 (0.943–1.281) | 0.225 | 19,854 | 1,435 | 18,419 |
| 3-year | HRS | Objective-only decline | 1.261 (1.101–1.443) | <0.001 | 19,854 | 1,435 | 18,419 |
| 3-year | HRS | Concordant decline | 1.642 (1.365–1.975) | <0.001 | 19,854 | 1,435 | 18,419 |
| 3-year | ELSA | Subjective-only decline | 1.672 (1.280–2.183) | <0.001 | 3,857 | 403 | 3,454 |
| 3-year | ELSA | Objective-only decline | 1.293 (0.985–1.697) | 0.064 | 3,857 | 403 | 3,454 |
| 3-year | ELSA | Concordant decline | 1.531 (1.033–2.267) | 0.034 | 3,857 | 403 | 3,454 |
| 3-year | SHARE | Subjective-only decline | 1.093 (0.956–1.251) | 0.194 | 24,571 | 1,739 | 22,832 |
| 3-year | SHARE | Objective-only decline | 1.060 (0.928–1.210) | 0.390 | 24,571 | 1,739 | 22,832 |
| 3-year | SHARE | Concordant decline | 1.358 (1.149–1.604) | <0.001 | 24,571 | 1,739 | 22,832 |
| 5-year | CHARLS | Subjective-only decline | 1.226 (1.074–1.400) | 0.003 | 14,047 | 2,452 | 11,595 |
| 5-year | CHARLS | Objective-only decline | 1.059 (0.954–1.176) | 0.282 | 14,047 | 2,452 | 11,595 |
| 5-year | CHARLS | Concordant decline | 1.528 (1.305–1.789) | <0.001 | 14,047 | 2,452 | 11,595 |
| 5-year | HRS | Subjective-only decline | 1.117 (1.000–1.248) | 0.050 | 19,854 | 2,708 | 17,146 |
| 5-year | HRS | Objective-only decline | 1.296 (1.175–1.430) | <0.001 | 19,854 | 2,708 | 17,146 |
| 5-year | HRS | Concordant decline | 1.609 (1.402–1.845) | <0.001 | 19,854 | 2,708 | 17,146 |
| 5-year | ELSA | Subjective-only decline | 1.672 (1.280–2.183) | <0.001 | 3,857 | 403 | 3,454 |
| 5-year | ELSA | Objective-only decline | 1.293 (0.985–1.697) | 0.064 | 3,857 | 403 | 3,454 |
| 5-year | ELSA | Concordant decline | 1.531 (1.033–2.267) | 0.034 | 3,857 | 403 | 3,454 |
| 5-year | SHARE | Subjective-only decline | 1.057 (0.940–1.188) | 0.357 | 24,571 | 2,303 | 22,268 |
| 5-year | SHARE | Objective-only decline | 1.084 (0.966–1.215) | 0.170 | 24,571 | 2,303 | 22,268 |
| 5-year | SHARE | Concordant decline | 1.374 (1.189–1.589) | <0.001 | 24,571 | 2,303 | 22,268 |

| **Panel C. Random-effects meta-analysis of fixed-window sensitivity analyses** | | | | | | | | | | |
| --- | --- | --- | --- | --- | --- | --- | --- | --- | --- | --- |
| **Window** | **Trajectory group** | **Pooled HR (95% CI)** | **P value** | **I², %** | **P value for heterogeneity** | **τ² (REML)** | **Total N** | **Total events** | **Cohorts included** |  |
| 3-year | Subjective-only decline | 1.241 (1.046–1.472) | 0.013 | 69.7 | 0.019 | 0.0220 | 62,329 | 5,066 | CHARLS, HRS, ELSA, SHARE |  |
| 3-year | Objective-only decline | 1.125 (0.994–1.273) | 0.062 | 60.2 | 0.057 | 0.0092 | 62,329 | 5,066 | CHARLS, HRS, ELSA, SHARE |  |
| 3-year | Concordant decline | 1.495 (1.343–1.665) | <0.001 | 0.0 | 0.509 | 0.0009 | 62,329 | 5,066 | CHARLS, HRS, ELSA, SHARE |  |
| 5-year | Subjective-only decline | 1.209 (1.029–1.421) | 0.021 | 72.0 | 0.013 | 0.0209 | 62,329 | 7,866 | CHARLS, HRS, ELSA, SHARE |  |
| 5-year | Objective-only decline | 1.161 (1.037–1.299) | 0.009 | 69.3 | 0.021 | 0.0083 | 62,329 | 7,866 | CHARLS, HRS, ELSA, SHARE |  |
| 5-year | Concordant decline | 1.504 (1.376–1.643) | <0.001 | 0.0 | 0.483 | 0.0009 | 62,329 | 7,866 | CHARLS, HRS, ELSA, SHARE |  |

| **Panel D. Variable audit for fixed-window analyses** | | | | | | | | |
| --- | --- | --- | --- | --- | --- | --- | --- | --- |
| **Cohort** | **Trajectory variable** | **Event variable** | **Time variable** | **Complete-case N** | **Events** | **Median follow-up** | **Maximum follow-up** |  |
| CHARLS | traj4_label | event | time_to_event | 14,047 | 3,378 | 5.00 | 7.25 |  |
| HRS | traj4_label | event | time_to_event | 19,854 | 6,808 | 7.83 | 18.92 |  |
| ELSA | traj4_label | event | time_to_event | 3,857 | 403 | 2.08 | 3.00 |  |
| SHARE | traj4_label | event | time_to_event | 24,571 | 3,548 | 6.33 | 7.08 |  |

**Notes:** Fixed-window analyses administratively truncated follow-up at 3 or 5 years after T1. Events occurring within the window were counted as incident functional dependence; events after the window were censored at the window boundary; participants without an event were censored at the earlier of the last observed follow-up or the window boundary. Models were adjusted for baseline age, sex, education, marital status, self-rated health, depressive symptoms, smoking, current drinking, baseline subjective memory, and baseline objective cognition. The reference category was concordant stable or improved. ELSA had a maximum observed follow-up of 3 years in the complete-case sample; therefore, the 5-year window did not additionally truncate ELSA follow-up and should be interpreted accordingly. PY indicates person-years; HR, hazard ratio; IQR, interquartile range; REML, restricted maximum likelihood.
