# Supplementary material for: Joint changes in subjective memory and objective cognition and risk of incident functional dependence: A harmonized analysis of four aging cohorts
Source: Alzheimers Dement. 2026 Aug 22;22(8):e71752. doi: 10.1002/alz.71752 (PMC13498894; doi:10.1002/alz.71752)
Supplement: Supplementary file 3 [file ALZ-22-e71752-s003.docx]

**Table S12. Sensitivity analysis excluding baseline subjective memory and baseline objective cognition from the fully adjusted model**

| **Panel A. Model definitions** | | |
| --- | --- | --- |
| **Model** | **Covariates** | **Purpose** |
| Primary fully adjusted model | age_t0, ragender_t0, education_3cat_t0, marry_bin_t0, srh_z_t0, depress_z_t0, smoke_ever_t0, drink_current_t0, sm_z_t0, cog_z_t0 | Prespecified primary model; adjusts for baseline subjective memory and baseline objective cognition |
| Reduced model without baseline SM/OC | age_t0, ragender_t0, education_3cat_t0, marry_bin_t0, srh_z_t0, depress_z_t0, smoke_ever_t0, drink_current_t0 | Sensitivity model excluding baseline subjective memory and baseline objective cognition while retaining the same complete-case sample |

| **Panel B. Cohort-specific reduced-model estimates and comparison with the primary fully adjusted model** | | | | | | | |
| --- | --- | --- | --- | --- | --- | --- | --- |
| **Cohort** | **Trajectory group** | **Primary HR** | **Reduced HR (95% CI)** | **Reduced P value** | **Change vs primary, %** | **N** | **Events** |
| CHARLS | Subjective-only decline | 1.257 | 0.996 (0.904–1.097) | 0.938 | -20.8 | 14,047 | 3,378 |
| CHARLS | Objective-only decline | 1.043 | 0.957 (0.877–1.043) | 0.316 | -8.3 | 14,047 | 3,378 |
| CHARLS | Concordant decline | 1.596 | 1.176 (1.042–1.328) | 0.009 | -26.3 | 14,047 | 3,378 |
| HRS | Subjective-only decline | 1.142 | 1.132 (1.062–1.207) | <0.001 | -0.9 | 19,854 | 6,808 |
| HRS | Objective-only decline | 1.178 | 1.091 (1.026–1.160) | 0.006 | -7.4 | 19,854 | 6,808 |
| HRS | Concordant decline | 1.464 | 1.327 (1.216–1.449) | <0.001 | -9.4 | 19,854 | 6,808 |
| ELSA | Subjective-only decline | 1.672 | 1.508 (1.178–1.930) | 0.001 | -9.8 | 3,857 | 403 |
| ELSA | Objective-only decline | 1.293 | 1.216 (0.931–1.589) | 0.152 | -5.9 | 3,857 | 403 |
| ELSA | Concordant decline | 1.531 | 1.296 (0.885–1.896) | 0.182 | -15.3 | 3,857 | 403 |
| SHARE | Subjective-only decline | 1.074 | 1.041 (0.956–1.134) | 0.358 | -3.1 | 24,571 | 3,548 |
| SHARE | Objective-only decline | 1.077 | 0.991 (0.905–1.085) | 0.839 | -8.0 | 24,571 | 3,548 |
| SHARE | Concordant decline | 1.383 | 1.244 (1.114–1.389) | <0.001 | -10.1 | 24,571 | 3,548 |

| **Panel C. Random-effects meta-analysis comparing primary and reduced models** | | | | | | | |
| --- | --- | --- | --- | --- | --- | --- | --- |
| **Trajectory** | **Primary pooled HR (95% CI)** | **Primary P value** | **Primary I²** | **Reduced pooled HR (95% CI)** | **Reduced P value** | **Reduced I²** | **Change vs primary, %** |
| Subjective-only decline | 1.222 (1.055–1.415) | 0.007 | 75.0% | 1.116 (0.975–1.276) | 0.110 | 75.9% | -8.7 |
| Objective-only decline | 1.116 (1.038–1.200) | 0.003 | 54.3% | 1.030 (0.952–1.114) | 0.466 | 63.7% | -7.7 |
| Concordant decline | 1.470 (1.381–1.566) | <0.001 | 0.0% | 1.262 (1.180–1.350) | <0.001 | 0.0% | -14.2 |

| **Panel D. Sample and variable audit** | | | | | | |
| --- | --- | --- | --- | --- | --- | --- |
| **Cohort** | **Outcome-eligible N** | **Analysis N** | **Events** | **Event variable** | **Time variable** | **Removed covariates** |
| CHARLS | 14,076 | 14,047 | 3,378 | event | time_to_event | sm_z_t0, cog_z_t0 |
| HRS | 19,995 | 19,854 | 6,808 | event | time_to_event | sm_z_t0, cog_z_t0 |
| ELSA | 4,591 | 3,857 | 403 | event | time_to_event | sm_z_t0, cog_z_t0 |
| SHARE | 25,045 | 24,571 | 3,548 | event | time_to_event | sm_z_t0, cog_z_t0 |

**Notes:** The reduced model used the same complete-case analytic sample as the primary fully adjusted model but excluded baseline subjective memory (sm_z_t0) and baseline objective cognition (cog_z_t0). All models retained the same trajectory exposure and reference category, concordant stable or improved. Primary HRs are shown for comparison with the prespecified fully adjusted model. Change vs primary was calculated as (reduced HR / primary HR − 1) × 100%. HR indicates hazard ratio; REML, restricted maximum likelihood; SM, subjective memory; OC, objective cognition.
