# Supplementary material for: Joint changes in subjective memory and objective cognition and risk of incident functional dependence: A harmonized analysis of four aging cohorts
Source: Alzheimers Dement. 2026 Aug 22;22(8):e71752. doi: 10.1002/alz.71752 (PMC13498894; doi:10.1002/alz.71752)
Supplement: Supplementary file 4 [file ALZ-22-e71752-s004.docx]

**Table S6. Age- and sex-adjusted associations between joint change patterns in subjective memory and objective cognition and incident functional dependence across the four cohorts**

| **Cohort** | **Trajectory group** | **HR (95% CI)** | **P value** | **N** | **Events** |
| --- | --- | --- | --- | --- | --- |
| **CHARLS** | Subjective-only decline | 0.884 (0.803–0.974) | 0.012 | 14,047 | 3,378 |
|  | Objective-only decline | 0.974 (0.893–1.062) | 0.552 | 14,047 | 3,378 |
|  | Concordant decline | 1.072 (0.950–1.211) | 0.258 | 14,047 | 3,378 |
| **HRS** | Subjective-only decline | 1.141 (1.070–1.216) | <0.001 | 19,854 | 6,808 |
|  | Objective-only decline | 1.128 (1.061–1.200) | <0.001 | 19,854 | 6,808 |
|  | Concordant decline | 1.365 (1.251–1.490) | <0.001 | 19,854 | 6,808 |
| **ELSA** | Subjective-only decline | 1.467 (1.147–1.877) | 0.002 | 3,857 | 403 |
|  | Objective-only decline | 1.120 (0.858–1.463) | 0.403 | 3,857 | 403 |
|  | Concordant decline | 1.219 (0.833–1.782) | 0.308 | 3,857 | 403 |
| **SHARE** | Subjective-only decline | 0.978 (0.898–1.065) | 0.603 | 24,571 | 3,548 |
|  | Objective-only decline | 1.032 (0.943–1.130) | 0.497 | 24,571 | 3,548 |
|  | Concordant decline | 1.227 (1.098–1.370) | <0.001 | 24,571 | 3,548 |

**Notes:** Values are hazard ratios (HRs) and 95% confidence intervals (CIs) derived from cohort-specific Cox proportional hazards models adjusted for baseline age and sex. The concordant stable or improved group served as the reference category. All analyses used the same final complete-case samples as the primary fully adjusted models.
