# Supplementary material for: Joint changes in subjective memory and objective cognition and risk of incident functional dependence: A harmonized analysis of four aging cohorts
Source: Alzheimers Dement. 2026 Aug 22;22(8):e71752. doi: 10.1002/alz.71752 (PMC13498894; doi:10.1002/alz.71752)
Supplement: Supplementary file 5 [file ALZ-22-e71752-s005.docx]

**Table S7. Schoenfeld residual-based diagnostics of the proportional hazards assumption for the primary trajectory terms in the fully adjusted cohort-specific Cox models**

| **Cohort** | **Variable** | **Schoenfeld test statistic** | **P value** | **Evidence of nonproportionality** |
| --- | --- | --- | --- | --- |
| **CHARLS** | Concordant decline | 0.299 | 0.585 | No |
|  | Objective-only decline | 0.127 | 0.721 | No |
|  | Subjective-only decline | 0.009 | 0.925 | No |
| **HRS** | Concordant decline | 7.462 | 0.006 | Yes |
|  | Objective-only decline | 11.517 | <0.001 | Yes |
|  | Subjective-only decline | 0.182 | 0.670 | No |
| **ELSA** | Concordant decline | 0.224 | 0.636 | No |
|  | Objective-only decline | 0.776 | 0.378 | No |
|  | Subjective-only decline | 0.209 | 0.647 | No |
| **SHARE** | Concordant decline | 0.896 | 0.344 | No |
|  | Objective-only decline | 0.015 | 0.902 | No |
|  | Subjective-only decline | 0.013 | 0.909 | No |

**Notes:** The proportional hazards assumption was assessed using Schoenfeld residual–based tests in each cohort-specific fully adjusted Cox model. P<0.05 indicated evidence of nonproportionality. The concordant stable or improved group served as the reference category. The fully adjusted models included baseline age, sex, education, marital status, self-rated health, depressive symptoms, smoking status, current drinking, baseline subjective memory, and baseline objective cognition.
