# Supplementary material for: Joint changes in subjective memory and objective cognition and risk of incident functional dependence: A harmonized analysis of four aging cohorts
Source: Alzheimers Dement. 2026 Aug 22;22(8):e71752. doi: 10.1002/alz.71752 (PMC13498894; doi:10.1002/alz.71752)
Supplement: Supplementary file 6 [file ALZ-22-e71752-s006.docx]

**Table S1. Harmonization of key variables across cohorts in the revised analysis**

| **Harmonized variable** | **CHARLS** | **HRS** | **ELSA** | **SHARE** | **Harmonization rule used in the revised analysis** |
| --- | --- | --- | --- | --- | --- |
| Subjective memory | `slfmem` at T0 and T1 | `slfmem` at T0 and T1 | `slfmem` at T0 and T1 | `slfmem` at T0 and T1 | Cohort-specific self-rated memory item at T0 and T1. Raw scores were recoded when necessary so that higher values indicated worse perceived memory and then standardized within cohort as `sm_z`. Change was defined as dSM = sm_z_t1 − sm_z_t0. Subjective memory decline was defined in the primary analysis as dSM ≥ 0.5 × SD(dSM); 0.3-SD and 0.7-SD thresholds were examined as sensitivity analyses. |
| Objective cognition | `tcog_z_z` → `cog_z` | `tcog_z_z` → `cog_z` | `tcog_z_z` → `cog_z` | Composite cognition score reconstructed from available memory and executive-function items (`tr20`, `orient`, `ser7`, and `verbf`) → `cog_z` | Objective cognition was standardized within cohort as `cog_z`. Change was defined as dOC = cog_z_t1 − cog_z_t0. Objective cognitive decline was defined in the primary analysis as dOC ≤ −0.5 × SD(dOC). In continuous change-score sensitivity analyses, the sign of dOC was reversed so that higher values consistently indicated greater objective-cognition worsening. |
| Trajectory group | Derived from dSM and dOC | Derived from dSM and dOC | Derived from dSM and dOC | Derived from dSM and dOC | Participants were classified into four mutually exclusive groups: concordant stable or improved, subjective-only decline, objective-only decline, and concordant decline. Concordant stable or improved served as the reference category. |
| Functional dependence | Available ADL/IADL count variables or item-level indicators, with `dependency` used for cross-checking where available | RAND/Harmonized HRS ADL/IADL count variables or item-level indicators, with `dependency` used for cross-checking where available | Available ADL/IADL count variables or item-level indicators, with `dependency` used for cross-checking where available | Item-level ADL/IADL indicators reconstructed into ADL and IADL counts, with `dependency` used for cross-checking where available | The primary harmonized cut-off in the revised analysis was ADL limitation count ≥1 or IADL limitation count ≥1 in each cohort. Participants with functional dependence at either T0 or T1 were excluded. Incident functional dependence was defined as the first newly observed functional dependence after T1. Detailed cohort-specific ADL/IADL items and cut-offs are provided in Table S13. |
| Alternative outcome definitions | — | — | ADL-only dependence sensitivity analysis | Stricter definition: ADL limitation count ≥1 or IADL limitation count ≥2 | Additional outcome-definition sensitivity analyses were performed for ELSA and SHARE because cohort-specific ADL/IADL item availability differed more clearly from the primary harmonized structure in these cohorts. Detailed estimates are provided in Table S2. |
| Self-rated health | `srh_t0` | `shlt_t0` | `shlt_t0` | `shlt_t0` | Self-rated health was harmonized so that higher values indicated poorer health and then standardized within cohort as `srh_z_t0` for the fully adjusted models. |
| Depressive symptoms | `cesd10_t0` | `cesd_t0` | `cesd_t0` | `eurod_t0` | Cohort-specific depressive symptom burden was harmonized using the available validated scale and standardized within cohort as `depress_z_t0`. |
| Smoking | `smokev_t0` | `smokev_t0` | `smokev_t0` | `smokev_t0` | Smoking was harmonized as an ever-smoking indicator (`smoke_ever_t0`) based on the cohort-specific baseline smoking-history variable. |
| Current drinking | `drinkl_t0` | `drink_t0` | `drink_t0` | `drinkxw_t0` | Alcohol use was harmonized as a current drinking indicator (`drink_current_t0`) using the cohort-specific alcohol-use variable retained in each analytic dataset. |
| Education | `raeducl_t0` | `raeducl_t0` | `raeducl_t0` | `raeducl_t0` | Education was harmonized using the baseline education variable and modeled as a categorical variable (`education_3cat_t0`) in the fully adjusted Cox models. |
| Marital status | `marry_t0` | `marry_t0` | `marry_t0` | `marry_t0` | Marital status was harmonized as a binary married/partnered indicator (`marry_bin_t0`) according to the cohort-specific baseline variable. |
| Sex | `ragender_t0` | `ragender_t0` | `ragender_t0` | `ragender_t0` | Sex was included as the baseline binary sex variable available in each cohort. |
| Age | `age_t0` | `age_t0` | `age_t0` | `age_t0` | Baseline age at T0 was included as a continuous covariate. |
| Baseline subjective memory | `sm_z_t0` | `sm_z_t0` | `sm_z_t0` | `sm_z_t0` | Baseline standardized subjective memory score was included in the primary fully adjusted model. A reduced-model sensitivity analysis excluding baseline subjective memory and baseline objective cognition was also conducted (Table S12). |
| Baseline objective cognition | `cog_z_t0` | `cog_z_t0` | `cog_z_t0` | `cog_z_t0` | Baseline standardized objective cognition score was included in the primary fully adjusted model. A reduced-model sensitivity analysis excluding baseline subjective memory and baseline objective cognition was also conducted (Table S12). |
| Follow-up time and event indicator | `time_to_event`, `event` | `time_to_event`, `event` | `time_to_event`, `event` | `time_to_event`, `event` | Follow-up began at T1. `event=1` indicated first observed functional dependence after T1. `time_to_event` was calculated from T1 to the first incident event or censoring at the last available post-T1 follow-up. |
| Death as competing event | Death status and timing available for competing-risk sensitivity analysis | Death status and timing available for competing-risk sensitivity analysis | Not used for competing-risk sensitivity analysis because mortality information did not adequately cover the analytic follow-up period | Death status and timing available for competing-risk sensitivity analysis | In CHARLS, HRS, and SHARE, death before observed functional dependence was coded as a competing event in Fine–Gray sensitivity analyses. ELSA was excluded from this sensitivity analysis because usable mortality follow-up was insufficient. |

**Notes:** Table S1 summarizes the updated cohort-specific source variables and harmonization rules used in the revised analytic pipeline across CHARLS, HRS, ELSA, and SHARE. T0 and T1 denote the first and second eligible observations with non-missing data on both subjective memory and objective cognition. Harmonization enhanced conceptual comparability across cohorts rather than strict identity of raw-item definitions. ADL, activities of daily living; IADL, instrumental activities of daily living; CHARLS, China Health and Retirement Longitudinal Study; HRS, Health and Retirement Study; ELSA, English Longitudinal Study of Ageing; SHARE, Survey of Health, Ageing and Retirement in Europe.
