# Supplementary material for: Joint changes in subjective memory and objective cognition and risk of incident functional dependence: A harmonized analysis of four aging cohorts
Source: Alzheimers Dement. 2026 Aug 22;22(8):e71752. doi: 10.1002/alz.71752 (PMC13498894; doi:10.1002/alz.71752)
Supplement: Supplementary file 7 [file ALZ-22-e71752-s007.docx]

**Table S10. Competing-risk sensitivity analyses treating death as a competing event**

| **Panel A. Competing-risk analytic samples** | | | | | |
| --- | --- | --- | --- | --- | --- |
| **Cohort** | **Risk-set definition** | **Final competing-risk sample, n** | **Functional dependence events** | **Competing deaths** | **Censored** |
| CHARLS | Observed post-T1 functional follow-up plus post-T1 deaths | 14,296 | 3,378 | 599 | 10,319 |
| HRS | Observed post-T1 functional follow-up plus ascertainable post-T1 deaths | 20,435 | 6,808 | 3,469 | 10,158 |
| SHARE | Primary exact-month competing-risk cohort | 25,485 | 3,559 | 831 | 21,095 |

| **Panel B. Cohort-specific Fine–Gray subdistribution hazards models** | | | | | | | |
| --- | --- | --- | --- | --- | --- | --- | --- |
| **Cohort** | **Trajectory group** | **sHR (95% CI)** | **P value** | **Functional dependence events** | **Competing deaths** | **Primary Cox HR** | **sHR change vs Cox HR, %** |
| CHARLS | Subjective-only decline | 1.258 (1.129–1.401) | <0.001 | 3,378 | 599 | 1.257 | 0.0 |
| CHARLS | Objective-only decline | 1.028 (0.941–1.123) | 0.545 | 3,378 | 599 | 1.043 | -1.5 |
| CHARLS | Concordant decline | 1.528 (1.341–1.740) | <0.001 | 3,378 | 599 | 1.596 | -4.3 |
| HRS | Subjective-only decline | 1.172 (1.092–1.257) | <0.001 | 6,808 | 3,469 | 1.142 | 2.6 |
| HRS | Objective-only decline | 1.121 (1.050–1.196) | <0.001 | 6,808 | 3,469 | 1.178 | -4.8 |
| HRS | Concordant decline | 1.367 (1.243–1.504) | <0.001 | 6,808 | 3,469 | 1.464 | -6.6 |
| SHARE | Subjective-only decline | 1.068 (0.973–1.172) | 0.166 | 3,559 | 831 | 1.074 | -0.6 |
| SHARE | Objective-only decline | 1.042 (0.950–1.143) | 0.384 | 3,559 | 831 | 1.077 | -3.3 |
| SHARE | Concordant decline | 1.294 (1.151–1.455) | <0.001 | 3,559 | 831 | 1.383 | -6.4 |

| **Panel C. Random-effects meta-analysis of Fine–Gray estimates** | | | | | | |
| --- | --- | --- | --- | --- | --- | --- |
| **Trajectory group** | **Pooled sHR (95% CI)** | **P value** | **I²** | **P value for heterogeneity** | **τ² (REML)** | **Cohorts included** |
| Subjective-only decline | 1.160 (1.065–1.265) | <0.001 | 61.9% | 0.073 | 0.0037 | CHARLS, HRS, SHARE |
| Objective-only decline | 1.071 (1.010–1.136) | 0.023 | 33.69% | 0.222 | 0.0010 | CHARLS, HRS, SHARE |
| Concordant decline | 1.386 (1.273–1.509) | <0.001 | 43.29% | 0.172 | 0.0023 | CHARLS, HRS, SHARE |

| **Panel D. ELSA mortality-data limitation** | | | | | |  |
| --- | --- | --- | --- | --- | --- | --- |
| **Cohort** | **Outcome-eligible N** | **Latest available death year** | **Latest analytic follow-up year** | **Fine–Gray analysis included** | **Reason** | |
| ELSA | 4,591 | 2012 | 2019 | No | Available mortality information did not cover the full analytic follow-up period and did not reliably identify post-T1 deaths in the outcome-eligible sample; therefore, coding all participants as free of the competing event would have been inappropriate. | |

**Notes:** Death before observed functional dependence was treated as a competing event. Event status was coded as 0 for censoring, 1 for incident functional dependence, and 2 for death before observed functional dependence. Fine–Gray subdistribution hazards models used the same covariate set as the primary fully adjusted Cox models, including baseline age, sex, education, marital status, self-rated health, depressive symptoms, smoking status, current drinking, baseline subjective memory, and baseline objective cognition. The concordant stable or improved group served as the reference category. Primary Cox HRs are shown for comparison with the corresponding Fine–Gray sHRs. ELSA was not included in the Fine–Gray analysis because mortality information did not adequately cover the analytic follow-up period. Abbreviations: CHARLS, China Health and Retirement Longitudinal Study; HRS, Health and Retirement Study; ELSA, English Longitudinal Study of Ageing; SHARE, Survey of Health, Ageing and Retirement in Europe; CI, confidence interval; HR, hazard ratio; REML, restricted maximum likelihood; sHR, subdistribution hazard ratio.
