# Supplementary material for: Joint changes in subjective memory and objective cognition and risk of incident functional dependence: A harmonized analysis of four aging cohorts
Source: Alzheimers Dement. 2026 Aug 22;22(8):e71752. doi: 10.1002/alz.71752 (PMC13498894; doi:10.1002/alz.71752)
Supplement: Supplementary file 8 [file ALZ-22-e71752-s008.docx]

**Table S4. Baseline characteristics by trajectory group in the final fully adjusted Cox samples**

| **Panel A. CHARLS** | | | | | |  |
| --- | --- | --- | --- | --- | --- | --- |
| **Characteristic** | **Concordant stable or improved** | **Subjective-only decline** | **Objective-only decline** | **Concordant decline** | **P value** |  |
| Participants, n | 7,500 | 2,468 | 2,895 | 1,184 | — |  |
| Age at T0, years | 54.69 ± 8.93 | 54.45 ± 8.95 | 55.90 ± 9.39 | 55.34 ± 9.46 | <0.001 |  |
| Self-rated health z-score | -0.07 ± 0.97 | 0.23 ± 1.04 | -0.08 ± 1.01 | 0.14 ± 1.01 | <0.001 |  |
| Depressive symptoms z-score | 0.04 ± 1.01 | -0.14 ± 0.94 | 0.07 ± 1.03 | -0.14 ± 0.92 | <0.001 |  |
| Baseline subjective memory z-score | 0.12 ± 0.84 | -1.24 ± 1.03 | 0.17 ± 0.87 | -1.15 ± 1.01 | <0.001 |  |
| Baseline objective cognition z-score | 0.09 ± 0.93 | 0.19 ± 0.88 | 0.52 ± 0.81 | 0.56 ± 0.77 | <0.001 |  |
| Women, n (%) | 3,838 (51.2%) | 1,259 (51.0%) | 1,405 (48.5%) | 576 (48.6%) | 0.054 |  |
| Married/partnered, n (%) | 6,534 (87.1%) | 2,153 (87.2%) | 2,485 (85.8%) | 1,009 (85.2%) | 0.117 |  |
| Ever smoking, n (%) | 3,027 (40.4%) | 982 (39.8%) | 1,194 (41.2%) | 452 (38.2%) | 0.313 |  |
| Current drinking, n (%) | 2,950 (39.3%) | 925 (37.5%) | 1,036 (35.8%) | 423 (35.7%) | 0.002 |  |
| Education: low, n (%) | 6,343 (84.6%) | 2,039 (82.6%) | 2,573 (88.9%) | 1,056 (89.2%) | <0.001 |  |
| Education: middle, n (%) | 948 (12.6%) | 360 (14.6%) | 280 (9.7%) | 111 (9.4%) | — |  |
| Education: high, n (%) | 209 (2.8%) | 69 (2.8%) | 42 (1.5%) | 17 (1.4%) | — |  |
| **Panel B. HRS** | | | | | |  |
| **Characteristic** | **Concordant stable or improved** | **Subjective-only decline** | **Objective-only decline** | **Concordant decline** | **P value** |  |
| Participants, n | 10,538 | 3,805 | 3,973 | 1,538 | — |  |
| Age at T0, years | 60.49 ± 9.79 | 59.80 ± 9.91 | 60.69 ± 10.12 | 60.36 ± 10.24 | <0.001 |  |
| Self-rated health z-score | 0.02 ± 0.98 | 0.04 ± 1.02 | -0.05 ± 1.00 | -0.07 ± 1.05 | <0.001 |  |
| Depressive symptoms z-score | -0.01 ± 0.99 | -0.02 ± 0.99 | 0.06 ± 1.05 | 0.01 ± 0.97 | <0.001 |  |
| Baseline subjective memory z-score | -0.08 ± 0.91 | -0.94 ± 0.84 | -0.03 ± 0.94 | -0.84 ± 0.86 | <0.001 |  |
| Baseline objective cognition z-score | 0.11 ± 0.81 | 0.01 ± 0.86 | 0.41 ± 0.77 | 0.34 ± 0.81 | <0.001 |  |
| Women, n (%) | 4,460 (42.3%) | 1,781 (46.8%) | 1,582 (39.8%) | 650 (42.3%) | <0.001 |  |
| Married/partnered, n (%) | 7,275 (69.0%) | 2,579 (67.8%) | 2,592 (65.2%) | 937 (60.9%) | <0.001 |  |
| Ever smoking, n (%) | 5,857 (55.6%) | 2,184 (57.4%) | 2,215 (55.8%) | 890 (57.9%) | 0.117 |  |
| Current drinking, n (%) | 6,286 (59.7%) | 2,365 (62.2%) | 2,255 (56.8%) | 867 (56.4%) | <0.001 |  |
| Education: low, n (%) | 1,652 (15.7%) | 648 (17.0%) | 843 (21.2%) | 359 (23.3%) | <0.001 |  |
| Education: middle, n (%) | 6,215 (59.0%) | 2,217 (58.3%) | 2,337 (58.8%) | 909 (59.1%) | — |  |
| Education: high, n (%) | 2,671 (25.3%) | 940 (24.7%) | 793 (20.0%) | 270 (17.6%) | — |  |
| **Panel C. ELSA** | | | | | |  |
| **Characteristic** | **Concordant stable or improved** | **Subjective-only decline** | **Objective-only decline** | **Concordant decline** | **P value** |  |
| Participants, n | 2,201 | 711 | 693 | 252 | — |  |
| Age at T0, years | 64.96 ± 7.83 | 65.63 ± 8.65 | 66.30 ± 8.54 | 66.40 ± 8.89 | <0.001 |  |
| Self-rated health z-score | 0.01 ± 0.99 | -0.01 ± 1.01 | 0.03 ± 0.98 | 0.03 ± 1.01 | 0.865 |  |
| Depressive symptoms z-score | -0.02 ± 1.00 | 0.01 ± 1.01 | -0.03 ± 0.98 | -0.06 ± 0.92 | 0.742 |  |
| Baseline subjective memory z-score | 0.01 ± 0.85 | -0.73 ± 0.85 | 0.09 ± 0.88 | -0.70 ± 0.91 | <0.001 |  |
| Baseline objective cognition z-score | 1.06 ± 0.53 | 0.97 ± 0.57 | 1.27 ± 0.46 | 1.24 ± 0.43 | <0.001 |  |
| Women, n (%) | 968 (44.0%) | 358 (50.4%) | 324 (46.8%) | 115 (45.6%) | 0.028 |  |
| Married/partnered, n (%) | 1,573 (71.5%) | 530 (74.5%) | 480 (69.3%) | 186 (73.8%) | 0.141 |  |
| Ever smoking, n (%) | 1,257 (57.1%) | 407 (57.2%) | 409 (59.0%) | 154 (61.1%) | 0.559 |  |
| Current drinking, n (%) | 2,013 (91.5%) | 643 (90.4%) | 629 (90.8%) | 230 (91.3%) | 0.840 |  |
| Education: low, n (%) | 386 (17.5%) | 122 (17.2%) | 149 (21.5%) | 60 (23.8%) | 0.082 |  |
| Education: middle, n (%) | 1,242 (56.4%) | 398 (56.0%) | 372 (53.7%) | 133 (52.8%) | — |  |
| Education: high, n (%) | 573 (26.0%) | 191 (26.9%) | 172 (24.8%) | 59 (23.4%) | — |  |
| **Panel D. SHARE** | | | | | |  |
| **Characteristic** | **Concordant stable or improved** | **Subjective-only decline** | **Objective-only decline** | **Concordant decline** | **P value** |  |
| Participants, n | 12,547 | 5,625 | 4,138 | 2,261 | — |  |
| Age at T0, years | 63.57 ± 8.91 | 63.42 ± 8.95 | 64.82 ± 8.92 | 64.61 ± 9.35 | <0.001 |  |
| Self-rated health z-score | -0.02 ± 0.99 | 0.11 ± 1.01 | -0.10 ± 1.00 | -0.00 ± 1.02 | <0.001 |  |
| Depressive symptoms z-score | 0.01 ± 1.01 | -0.05 ± 0.97 | 0.04 ± 1.02 | -0.03 ± 0.99 | <0.001 |  |
| Baseline subjective memory z-score | 0.14 ± 0.85 | -0.78 ± 0.83 | 0.22 ± 0.82 | -0.70 ± 0.86 | <0.001 |  |
| Baseline objective cognition z-score | 0.12 ± 0.68 | 0.13 ± 0.67 | 0.40 ± 0.65 | 0.36 ± 0.66 | <0.001 |  |
| Women, n (%) | 5,298 (42.2%) | 2,447 (43.5%) | 1,742 (42.1%) | 954 (42.2%) | 0.382 |  |
| Married/partnered, n (%) | 8,823 (70.3%) | 3,957 (70.3%) | 2,888 (69.8%) | 1,598 (70.7%) | 0.882 |  |
| Ever smoking, n (%) | 5,918 (47.2%) | 2,644 (47.0%) | 1,912 (46.2%) | 973 (43.0%) | 0.003 |  |
| Current drinking, n (%) | 6,515 (51.9%) | 2,804 (49.8%) | 2,067 (50.0%) | 1,051 (46.5%) | <0.001 |  |
| Education: low, n (%) | 4,296 (34.2%) | 1,784 (31.7%) | 1,643 (39.7%) | 954 (42.2%) | <0.001 |  |
| Education: middle, n (%) | 5,118 (40.8%) | 2,365 (42.0%) | 1,619 (39.1%) | 841 (37.2%) | — |  |
| Education: high, n (%) | 3,133 (25.0%) | 1,476 (26.2%) | 876 (21.2%) | 466 (20.6%) | — |  |
| **Notes:** Continuous variables are presented as mean ± standard deviation and were compared using one-way analysis of variance. Categorical variables are presented as n (%) and were compared using chi-square tests. All characteristics were recalculated in the cohort-specific final complete-case samples used in the fully adjusted Cox models. | | | | | |  |
|  |  |  |  |  |  |  |
|  |  |  |  |  |  |  |
