# Supplementary material for: Joint changes in subjective memory and objective cognition and risk of incident functional dependence: A harmonized analysis of four aging cohorts
Source: Alzheimers Dement. 2026 Aug 22;22(8):e71752. doi: 10.1002/alz.71752 (PMC13498894; doi:10.1002/alz.71752)
Supplement: Supplementary file 9 [file ALZ-22-e71752-s009.docx]

**Table S14. Cohort-specific wording and coding of subjective memory items**

| **Cohort** | **Harmonized variable used in analysis** | **Original item / documentation source** | **Original or master English question wording** | **Response options** | **Coding used for harmonization** | **Comparability note** |
| --- | --- | --- | --- | --- | --- | --- |
| CHARLS | `slfmem` at T0 and T1 | CHARLS cognitive module, item DC006 | “How would you rate your memory at the present time? Would you say it is excellent, very good, good, fair or poor?” | 1=Excellent; 2=Very good; 3=Good; 4=Fair; 5=Poor | Higher values already indicated poorer perceived memory; item was standardized within cohort as `sm_z`. | English wording and response scale closely match the HRS/SHARE memory-rating item. |
| HRS | `slfmem` at T0 and T1 | HRS/RAND cognitive documentation; self-rated memory, present | For most waves: “How would you rate your memory at the present time? Would you say it is excellent, very good, good, fair, or poor?” | 1=Excellent; 2=Very good; 3=Good; 4=Fair; 5=Poor | Higher values indicated poorer perceived memory; item was standardized within cohort as `sm_z`. | The harmonized variable was used consistently in the analytic pipeline. |
| ELSA | `slfmem` at T0 and T1 | ELSA cognitive function module, item CFMETM / CfMetM | “Part of this study is concerned with people’s memory. How would you rate your memory at the present time? Would you say it is excellent, very good, good, fair, or poor?” In later interviewer questionnaires, the item is introduced as concerning memory and ability to think about things, followed by: “First, how would you rate your memory at the present time?” | 1=Excellent; 2=Very good; 3=Good; 4=Fair; 5=Poor | Higher values indicated poorer perceived memory; item was standardized within cohort as `sm_z`. | The response scale was comparable with CHARLS/HRS/SHARE, but wording/context differed slightly because the item was embedded in the ELSA cognitive-function module. |
| SHARE | `slfmem` at T0 and T1 | SHARE cognitive function module, self-rated memory item CF841 | Master English wording: “First, how would you rate [respondent name]’s memory at the present time? Would you say it is excellent, very good, good, fair or poor?” | 1=Excellent; 2=Very good; 3=Good; 4=Fair; 5=Poor | Higher values indicated poorer perceived memory; item was standardized within cohort as `sm_z`. | SHARE is administered in multiple countries and languages; the harmonized analysis used the comparable self-rated memory construct, while acknowledging potential cross-language and cultural response differences. |

**Notes:** T0 and T1 denote the first and second eligible observations with non-missing subjective memory and objective cognition data. All four cohorts used a five-level self-rated memory item with higher values indicating poorer perceived memory after harmonization. The original wording is shown to clarify comparability across cohorts; residual differences in wording, survey context, language, and cultural response style were acknowledged as potential sources of measurement heterogeneity. CHARLS, China Health and Retirement Longitudinal Study; HRS, Health and Retirement Study; ELSA, English Longitudinal Study of Ageing; SHARE, Survey of Health, Ageing and Retirement in Europe.
