# Supplementary material for: Joint changes in subjective memory and objective cognition and risk of incident functional dependence: A harmonized analysis of four aging cohorts
Source: Alzheimers Dement. 2026 Aug 22;22(8):e71752. doi: 10.1002/alz.71752 (PMC13498894; doi:10.1002/alz.71752)
Supplement: Supplementary file 10 [file ALZ-22-e71752-s010.docx]

**Table S13. Cohort-specific operationalization of functional dependence**

| **Cohort** | **Source information used** | **ADL items considered** | **IADL items considered** | **Primary cut-off for functional dependence** | **Baseline exclusion rule** | **Incident event definition after T1** | **Sensitivity analysis / notes** |
| --- | --- | --- | --- | --- | --- | --- | --- |
| CHARLS | Harmonized ADL/IADL count variables or item-level difficulty/help indicators | Dressing; bathing/showering; eating; getting in/out of bed; using the toilet; continence/control of urination or defecation | Doing household chores; preparing/cooking meals; shopping; managing money; taking medications; using the telephone | ADL limitation count ≥1 or IADL limitation count ≥1 | Participants with functional dependence at T0 or T1 were excluded | First post-T1 wave at which ADL limitation count ≥1 or IADL limitation count ≥1 was observed | Primary analysis |
| HRS | RAND/Harmonized HRS ADL/IADL count variables or item-level difficulty/help indicators | Dressing; bathing/showering; eating; getting in/out of bed; using the toilet; walking across a room | Preparing hot meals; shopping for groceries; making telephone calls; taking medications; managing money | ADL limitation count ≥1 or IADL limitation count ≥1 | Participants with functional dependence at T0 or T1 were excluded | First post-T1 wave at which ADL limitation count ≥1 or IADL limitation count ≥1 was observed | Primary analysis |
| ELSA | Harmonized ELSA ADL/IADL count variables or item-level difficulty/help indicators | Dressing; walking across a room; bathing/showering; eating/cutting up food; getting in/out of bed; using the toilet | Using a map; preparing a hot meal; shopping for groceries; making telephone calls; taking medications; doing work around the house or garden; managing money | ADL limitation count ≥1 or IADL limitation count ≥1 | Participants with functional dependence at T0 or T1 were excluded | First post-T1 wave at which ADL limitation count ≥1 or IADL limitation count ≥1 was observed | Because outcome operationalization differed more clearly from the primary harmonized ADL/IADL structure |
| SHARE | Harmonized SHARE ADL/IADL count variables or item-level difficulty/help indicators | Dressing; walking across a room; bathing/showering; eating/cutting up food; getting in/out of bed; using the toilet | Using a map; preparing a hot meal; shopping for groceries; making telephone calls; taking medications; doing work around the house or garden; managing money | ADL limitation count ≥1 or IADL limitation count ≥1 | Participants with functional dependence at T0 or T1 were excluded | First post-T1 wave at which ADL limitation count ≥1 or IADL limitation count ≥1 was observed | A stricter outcome definition of ADL limitation count ≥1 or IADL limitation count ≥2 was examined as a sensitivity analysis |

**Notes:** Functional dependence was harmonized at the conceptual level across cohorts. The primary outcome was defined as any ADL or IADL limitation after T1, with participants dependent at T0 or T1 excluded. When an established harmonized ADL/IADL count variable was available, the count was used; otherwise, cohort-specific item-level indicators were combined to construct comparable counts. ADL denotes activities of daily living; IADL denotes instrumental activities of daily living; T0, baseline; T1, first follow-up used for trajectory derivation.
