# Supplementary material for: Joint changes in subjective memory and objective cognition and risk of incident functional dependence: A harmonized analysis of four aging cohorts
Source: Alzheimers Dement. 2026 Aug 22;22(8):e71752. doi: 10.1002/alz.71752 (PMC13498894; doi:10.1002/alz.71752)
Supplement: Supplementary file 11 [file ALZ-22-e71752-s012.docx]

**Table S2. Additional sensitivity analyses using alternative outcome definitions in ELSA and SHARE**

| **Section** | **Sensitivity analysis** | **Trajectory group** | **ELSA HR (95% CI), P value** | **SHARE HR (95% CI), P value** |
| --- | --- | --- | --- | --- |
| Alternative outcome definition in ELSA | ELSA ADL-only outcome | Subjective-only decline | 0.905 (0.631–1.298), P=0.588 | — |
| Alternative outcome definition in ELSA | ELSA ADL-only outcome | Objective-only decline | 0.722 (0.487–1.072), P=0.106 | — |
| Alternative outcome definition in ELSA | ELSA ADL-only outcome | Concordant decline | 0.574 (0.296–1.113), P=0.100 | — |
| Alternative outcome definition in SHARE | SHARE ADL limitation count ≥1 or IADL limitation count ≥2 | Subjective-only decline | — | 1.102 (0.945–1.286), P=0.217 |
| Alternative outcome definition in SHARE | SHARE ADL limitation count ≥1 or IADL limitation count ≥2 | Objective-only decline | — | 1.013 (0.870–1.179), P=0.873 |
| Alternative outcome definition in SHARE | SHARE ADL limitation count ≥1 or IADL limitation count ≥2 | Concordant decline | — | 1.038 (0.850–1.267), P=0.715 |

**Notes:** The primary harmonized outcome definition was ADL limitation count ≥1 or IADL limitation count ≥1 in each cohort, as detailed in Table S13. This table reports additional sensitivity analyses using alternative outcome definitions in cohorts where outcome operationalization differed more clearly from the primary harmonized definition. In ELSA, the alternative outcome was restricted to ADL-only dependence. In SHARE, functional dependence was alternatively defined using a stricter threshold of ADL limitation count ≥1 or IADL limitation count ≥2. Estimates are HRs and 95% CIs from fully adjusted Cox models, with the concordant stable or improved group as the reference. An em dash indicates that the corresponding alternative outcome analysis was not conducted in that cohort.
