# Supplementary material for: Joint changes in subjective memory and objective cognition and risk of incident functional dependence: A harmonized analysis of four aging cohorts
Source: Alzheimers Dement. 2026 Aug 22;22(8):e71752. doi: 10.1002/alz.71752 (PMC13498894; doi:10.1002/alz.71752)
Supplement: Supplementary file 12 [file ALZ-22-e71752-s013.docx]

**Table S8. Baseline characteristics of participants included in and excluded from the final outcome analyses**

| **Panel A. Final complete-case participants versus all participants not included in the final complete-case outcome analysis** | | | | | | | | |
| --- | --- | --- | --- | --- | --- | --- | --- | --- |
| **Cohort** | **Characteristic** | **Final complete-case participants** | | **All excluded from final outcome analysis** | | |  | |
|  |  | **Summary** | **Missing, n (%)** | **Summary** | **Missing, n (%)** | | | **\|SMD\|** |
| **CHARLS (Final complete-case participants: n=14,047; All excluded from final outcome analysis: n=1,761)** | | | | | | | | |
| CHARLS | Age at T0, years | 54.95 ± 9.09 | 0 (0.0%) | 54.61 ± 10.05 | | 15 (0.9%) | 0.035 | |
|  | Women, n (%) | 7,078 (50.4%) | 0 (0.0%) | 939 (53.3%) | | 0 (0.0%) | 0.059 | |
|  | Education: low, n (%) | 12,011 (85.5%) | 0 (0.0%) | 1,370 (77.8%) | | 0 (0.0%) | 0.200 | |
|  | Education: middle, n (%) | 1,699 (12.1%) | 0 (0.0%) | 277 (15.7%) | | 0 (0.0%) | 0.105 | |
|  | Education: high, n (%) | 337 (2.4%) | 0 (0.0%) | 114 (6.5%) | | 0 (0.0%) | 0.199 | |
|  | Self-rated health z-score | 3.24 ± 0.88 | 0 (0.0%) | 3.37 ± 0.95 | | 2 (0.1%) | 0.146 | |
|  | Ever smoking, n (%) | 5,655 (40.3%) | 0 (0.0%) | 723 (41.1%) | | 1 (0.1%) | 0.017 | |
|  | Current drinking, n (%) | 5,334 (38.0%) | 0 (0.0%) | 749 (42.6%) | | 4 (0.2%) | 0.095 | |
|  | Baseline subjective memory z-score | -0.22 ± 1.07 | 0 (0.0%) | -0.42 ± 1.11 | | 0 (0.0%) | 0.182 | |
|  | Baseline objective cognition z-score | 0.23 ± 0.90 | 0 (0.0%) | 0.36 ± 0.93 | | 0 (0.0%) | 0.132 | |
| **HRS (Final complete-case participants: n=19,854; All excluded from final outcome analysis: n=1,299)** | | | | | | | | |
| HRS | Age at T0, years | 60.39 ± 9.92 | 0 (0.0%) | 62.85 ± 11.86 | | 0 (0.0%) | 0.225 | |
|  | Women, n (%) | 8,473 (42.7%) | 0 (0.0%) | 684 (52.7%) | | 0 (0.0%) | 0.201 | |
|  | Education: low, n (%) | 3,502 (17.6%) | 0 (0.0%) | 260 (20.1%) | | 5 (0.4%) | 0.063 | |
|  | Education: middle, n (%) | 11,678 (58.8%) | 0 (0.0%) | 755 (58.3%) | | 5 (0.4%) | 0.010 | |
|  | Education: high, n (%) | 4,674 (23.5%) | 0 (0.0%) | 279 (21.6%) | | 5 (0.4%) | 0.047 | |
|  | Ever smoking, n (%) | 11,146 (56.1%) | 0 (0.0%) | 734 (62.2%) | | 119 (9.2%) | 0.124 | |
|  | Baseline subjective memory z-score | -0.29 ± 0.97 | 0 (0.0%) | -0.28 ± 0.99 | | 0 (0.0%) | 0.007 | |
|  | Baseline objective cognition z-score | 0.17 ± 0.83 | 0 (0.0%) | 0.02 ± 0.88 | | 0 (0.0%) | 0.174 | |
| **ELSA (Final complete-case participants: n=3,857; All excluded from final outcome analysis: n=1,641)** | | | | | | | | |
| ELSA | Age at T0, years | 65.42 ± 8.21 | 0 (0.0%) | 64.42 ± 9.74 | | 0 (0.0%) | 0.111 | |
|  | Women, n (%) | 1,765 (45.8%) | 0 (0.0%) | 709 (43.2%) | | 0 (0.0%) | 0.051 | |
|  | Education: low, n (%) | 717 (18.6%) | 0 (0.0%) | 282 (24.5%) | | 492 (30.0%) | 0.145 | |
|  | Education: middle, n (%) | 2,145 (55.6%) | 0 (0.0%) | 621 (54.0%) | | 492 (30.0%) | 0.031 | |
|  | Education: high, n (%) | 995 (25.8%) | 0 (0.0%) | 246 (21.4%) | | 492 (30.0%) | 0.103 | |
|  | Ever smoking, n (%) | 2,227 (57.7%) | 0 (0.0%) | 963 (58.8%) | | 3 (0.2%) | 0.021 | |
|  | Baseline subjective memory z-score | -0.16 ± 0.92 | 0 (0.0%) | -0.13 ± 0.97 | | 0 (0.0%) | 0.033 | |
|  | Baseline objective cognition z-score | 1.09 ± 0.53 | 0 (0.0%) | 1.00 ± 0.59 | | 0 (0.0%) | 0.174 | |
| **SHARE (Final complete-case participants: n=24,571; All excluded from final outcome analysis: n=19,532)** | | | | | | | | |
| SHARE | Age at T0, years | 63.84 ± 8.98 | 0 (0.0%) | 62.31 ± 9.02 | | 0 (0.0%) | 0.170 | |
|  | Women, n (%) | 10,441 (42.5%) | 0 (0.0%) | 8,799 (45.0%) | | 0 (0.0%) | 0.052 | |
|  | Education: low, n (%) | 8,677 (35.3%) | 0 (0.0%) | 6,530 (33.4%) | | 0 (0.0%) | 0.040 | |
|  | Education: middle, n (%) | 9,943 (40.5%) | 0 (0.0%) | 7,913 (40.5%) | | 0 (0.0%) | 0.001 | |
|  | Education: high, n (%) | 5,951 (24.2%) | 0 (0.0%) | 5,089 (26.1%) | | 0 (0.0%) | 0.042 | |
|  | Ever smoking, n (%) | 11,447 (46.6%) | 0 (0.0%) | 9,363 (49.3%) | | 528 (2.7%) | 0.054 | |
|  | Baseline subjective memory z-score | -0.13 ± 0.94 | 0 (0.0%) | -0.27 ± 0.95 | | 0 (0.0%) | 0.146 | |
|  | Baseline objective cognition z-score | 0.19 ± 0.68 | 0 (0.0%) | 0.19 ± 0.65 | | 0 (0.0%) | 0.010 | |

| **Panel B. Outcome-eligible participants versus participants who were outcome-ineligible or lacked valid post-T1 follow-up** | | | | | | |
| --- | --- | --- | --- | --- | --- | --- |
| **Cohort** | **Characteristic** | **Outcome-eligible participants** | | **Outcome-ineligible or no valid post-T1 follow-up** | |  |
|  |  | **Summary** | **Missing, n (%)** | **Summary** | **Missing, n (%)** | **\|SMD\|** |
| **CHARLS (Outcome-eligible participants: n=14,076; Outcome-ineligible or no valid post-T1 follow-up: n=1,732)** | | | | | | |
| CHARLS | Age at T0, years | 54.95 ± 9.09 | 7 (0.0%) | 54.58 ± 10.05 | 8 (0.5%) | 0.039 |
|  | Women, n (%) | 7,093 (50.4%) | 0 (0.0%) | 924 (53.3%) | 0 (0.0%) | 0.059 |
|  | Education: low, n (%) | 12,036 (85.5%) | 0 (0.0%) | 1,345 (77.7%) | 0 (0.0%) | 0.204 |
|  | Education: middle, n (%) | 1,703 (12.1%) | 0 (0.0%) | 273 (15.8%) | 0 (0.0%) | 0.106 |
|  | Education: high, n (%) | 337 (2.4%) | 0 (0.0%) | 114 (6.6%) | 0 (0.0%) | 0.203 |
|  | Self-rated health z-score | 3.24 ± 0.88 | 2 (0.0%) | 3.38 ± 0.95 | 0 (0.0%) | 0.154 |
|  | Ever smoking, n (%) | 5,666 (40.3%) | 1 (0.0%) | 712 (41.1%) | 0 (0.0%) | 0.017 |
|  | Current drinking, n (%) | 5,342 (38.0%) | 4 (0.0%) | 741 (42.8%) | 0 (0.0%) | 0.098 |
|  | Baseline subjective memory z-score | -0.22 ± 1.07 | 0 (0.0%) | -0.42 ± 1.11 | 0 (0.0%) | 0.187 |
|  | Baseline objective cognition z-score | 0.23 ± 0.90 | 0 (0.0%) | 0.36 ± 0.93 | 0 (0.0%) | 0.142 |
| **HRS (Outcome-eligible participants: n=19,995; Outcome-ineligible or no valid post-T1 follow-up: n=1,158)** | | | | | | |
| HRS | Age at T0, years | 60.40 ± 9.92 | 0 (0.0%) | 62.92 ± 12.03 | 0 (0.0%) | 0.228 |
|  | Women, n (%) | 8,536 (42.7%) | 0 (0.0%) | 621 (53.6%) | 0 (0.0%) | 0.220 |
|  | Education: low, n (%) | 3,540 (17.7%) | 4 (0.0%) | 222 (19.2%) | 1 (0.1%) | 0.038 |
|  | Education: middle, n (%) | 11,747 (58.8%) | 4 (0.0%) | 686 (59.3%) | 1 (0.1%) | 0.011 |
|  | Education: high, n (%) | 4,704 (23.5%) | 4 (0.0%) | 249 (21.5%) | 1 (0.1%) | 0.048 |
|  | Ever smoking, n (%) | 11,166 (56.2%) | 113 (0.6%) | 714 (62.0%) | 6 (0.5%) | 0.119 |
|  | Baseline subjective memory z-score | -0.29 ± 0.97 | 0 (0.0%) | -0.30 ± 0.99 | 0 (0.0%) | 0.008 |
|  | Baseline objective cognition z-score | 0.17 ± 0.83 | 0 (0.0%) | 0.02 ± 0.85 | 0 (0.0%) | 0.177 |
| **ELSA (Outcome-eligible participants: n=4,591; Outcome-ineligible or no valid post-T1 follow-up: n=907)** | | | | | | |
| ELSA | Age at T0, years | 65.16 ± 8.54 | 0 (0.0%) | 64.90 ± 9.48 | 0 (0.0%) | 0.030 |
|  | Women, n (%) | 2,066 (45.0%) | 0 (0.0%) | 408 (45.0%) | 0 (0.0%) | 0.000 |
|  | Education: low, n (%) | 810 (19.4%) | 405 (8.8%) | 189 (23.0%) | 87 (9.6%) | 0.091 |
|  | Education: middle, n (%) | 2,315 (55.3%) | 405 (8.8%) | 451 (55.0%) | 87 (9.6%) | 0.006 |
|  | Education: high, n (%) | 1,061 (25.3%) | 405 (8.8%) | 180 (22.0%) | 87 (9.6%) | 0.080 |
|  | Ever smoking, n (%) | 2,632 (57.3%) | 1 (0.0%) | 558 (61.7%) | 2 (0.2%) | 0.088 |
|  | Baseline subjective memory z-score | -0.16 ± 0.93 | 0 (0.0%) | -0.09 ± 0.96 | 0 (0.0%) | 0.068 |
|  | Baseline objective cognition z-score | 1.08 ± 0.53 | 0 (0.0%) | 0.97 ± 0.61 | 0 (0.0%) | 0.188 |
| **SHARE (Outcome-eligible participants: n=25,045; Outcome-ineligible or no valid post-T1 follow-up: n=19,058)** | | | | | | |
| SHARE | Age at T0, years | 63.84 ± 8.99 | 0 (0.0%) | 62.28 ± 9.01 | 0 (0.0%) | 0.174 |
|  | Women, n (%) | 10,643 (42.5%) | 0 (0.0%) | 8,597 (45.1%) | 0 (0.0%) | 0.053 |
|  | Education: low, n (%) | 8,875 (35.4%) | 0 (0.0%) | 6,332 (33.2%) | 0 (0.0%) | 0.047 |
|  | Education: middle, n (%) | 10,121 (40.4%) | 0 (0.0%) | 7,735 (40.6%) | 0 (0.0%) | 0.004 |
|  | Education: high, n (%) | 6,049 (24.2%) | 0 (0.0%) | 4,991 (26.2%) | 0 (0.0%) | 0.047 |
|  | Ever smoking, n (%) | 11,536 (46.7%) | 360 (1.4%) | 9,274 (49.1%) | 168 (0.9%) | 0.047 |
|  | Baseline subjective memory z-score | -0.13 ± 0.94 | 0 (0.0%) | -0.28 ± 0.95 | 0 (0.0%) | 0.158 |
|  | Baseline objective cognition z-score | 0.19 ± 0.68 | 0 (0.0%) | 0.19 ± 0.64 | 0 (0.0%) | 0.001 |

| **Panel C. Complete-case versus covariate-incomplete participants among those eligible for the outcome analysis** | | | | | | |  |
| --- | --- | --- | --- | --- | --- | --- | --- |
| **Cohort** | **Characteristic** | **Complete-case participants** | | **Covariate-incomplete participants** | |  |  |
|  |  | **Summary** | **Missing, n (%)** | **Summary** | **Missing, n (%)** | **\|SMD\|** |  |
| **CHARLS (Complete-case participants: n=14,047; Covariate-incomplete participants: n=29)** | | | | | | |  |
| CHARLS | Age at T0, years | 54.95 ± 9.09 | 0 (0.0%) | 56.82 ± 10.41 | 7 (24.1%) | 0.191 |  |
|  | Women, n (%) | 7,078 (50.4%) | 0 (0.0%) | 15 (51.7%) | 0 (0.0%) | 0.027 |  |
|  | Education: low, n (%) | 12,011 (85.5%) | 0 (0.0%) | 25 (86.2%) | 0 (0.0%) | 0.020 |  |
|  | Education: middle, n (%) | 1,699 (12.1%) | 0 (0.0%) | 4 (13.8%) | 0 (0.0%) | 0.051 |  |
|  | Education: high, n (%) | 337 (2.4%) | 0 (0.0%) | 0 (0.0%) | 0 (0.0%) | 0.222 |  |
|  | Married/partnered, n (%) | 12,181 (86.7%) | 0 (0.0%) | 26 (89.7%) | 0 (0.0%) | 0.091 |  |
|  | Self-rated health z-score | 0.00 ± 1.00 | 0 (0.0%) | -0.31 ± 1.19 | 2 (6.9%) | 0.283 |  |
|  | Depressive symptoms z-score | -0.00 ± 1.00 | 0 (0.0%) | 0.25 ± 1.25 | 15 (51.7%) | 0.218 |  |
|  | Ever smoking, n (%) | 5,655 (40.3%) | 0 (0.0%) | 11 (39.3%) | 1 (3.4%) | 0.020 |  |
|  | Current drinking, n (%) | 5,334 (38.0%) | 0 (0.0%) | 8 (32.0%) | 4 (13.8%) | 0.125 |  |
|  | Baseline subjective memory z-score | -0.22 ± 1.07 | 0 (0.0%) | -0.13 ± 1.07 | 0 (0.0%) | 0.081 |  |
|  | Baseline objective cognition z-score | 0.23 ± 0.90 | 0 (0.0%) | -0.15 ± 1.06 | 0 (0.0%) | 0.388 |  |
| **HRS (Complete-case participants: n=19,854; Covariate-incomplete participants: n=141)** | | | | | | |  |
| HRS | Age at T0, years | 60.39 ± 9.92 | 0 (0.0%) | 62.28 ± 10.40 | 0 (0.0%) | 0.186 |  |
|  | Women, n (%) | 8,473 (42.7%) | 0 (0.0%) | 63 (44.7%) | 0 (0.0%) | 0.040 |  |
|  | Education: low, n (%) | 3,502 (17.6%) | 0 (0.0%) | 38 (27.7%) | 4 (2.8%) | 0.243 |  |
|  | Education: middle, n (%) | 11,678 (58.8%) | 0 (0.0%) | 69 (50.4%) | 4 (2.8%) | 0.170 |  |
|  | Education: high, n (%) | 4,674 (23.5%) | 0 (0.0%) | 30 (21.9%) | 4 (2.8%) | 0.039 |  |
|  | Married/partnered, n (%) | 13,383 (67.4%) | 0 (0.0%) | 97 (71.9%) | 6 (4.3%) | 0.097 |  |
|  | Self-rated health z-score | -0.00 ± 1.00 | 0 (0.0%) | 0.13 ± 0.92 | 7 (5.0%) | 0.135 |  |
|  | Depressive symptoms z-score | 0.00 ± 1.00 | 0 (0.0%) | -0.07 ± 0.93 | 10 (7.1%) | 0.070 |  |
|  | Ever smoking, n (%) | 11,146 (56.1%) | 0 (0.0%) | 20 (71.4%) | 113 (80.1%) | 0.322 |  |
|  | Current drinking, n (%) | 11,773 (59.3%) | 0 (0.0%) | 64 (46.4%) | 3 (2.1%) | 0.261 |  |
|  | Baseline subjective memory z-score | -0.29 ± 0.97 | 0 (0.0%) | -0.17 ± 0.97 | 0 (0.0%) | 0.122 |  |
|  | Baseline objective cognition z-score | 0.17 ± 0.83 | 0 (0.0%) | 0.03 ± 1.06 | 0 (0.0%) | 0.147 |  |
| **ELSA (Complete-case participants: n=3,857; Covariate-incomplete participants: n=734)** | | | | | | |  |
| ELSA | Age at T0, years | 65.42 ± 8.21 | 0 (0.0%) | 63.83 ± 10.04 | 0 (0.0%) | 0.173 |  |
|  | Women, n (%) | 1,765 (45.8%) | 0 (0.0%) | 301 (41.0%) | 0 (0.0%) | 0.096 |  |
|  | Education: low, n (%) | 717 (18.6%) | 0 (0.0%) | 93 (28.3%) | 405 (55.2%) | 0.230 |  |
|  | Education: middle, n (%) | 2,145 (55.6%) | 0 (0.0%) | 170 (51.7%) | 405 (55.2%) | 0.079 |  |
|  | Education: high, n (%) | 995 (25.8%) | 0 (0.0%) | 66 (20.1%) | 405 (55.2%) | 0.137 |  |
|  | Married/partnered, n (%) | 2,769 (71.8%) | 0 (0.0%) | 477 (65.1%) | 1 (0.1%) | 0.145 |  |
|  | Self-rated health z-score | 0.01 ± 0.99 | 0 (0.0%) | -0.05 ± 1.04 | 1 (0.1%) | 0.060 |  |
|  | Depressive symptoms z-score | -0.02 ± 0.99 | 0 (0.0%) | 0.11 ± 1.04 | 8 (1.1%) | 0.127 |  |
|  | Ever smoking, n (%) | 2,227 (57.7%) | 0 (0.0%) | 405 (55.3%) | 1 (0.1%) | 0.050 |  |
|  | Current drinking, n (%) | 3,515 (91.1%) | 0 (0.0%) | 352 (92.1%) | 352 (48.0%) | 0.037 |  |
|  | Baseline subjective memory z-score | -0.16 ± 0.92 | 0 (0.0%) | -0.17 ± 0.97 | 0 (0.0%) | 0.009 |  |
|  | Baseline objective cognition z-score | 1.09 ± 0.53 | 0 (0.0%) | 1.02 ± 0.57 | 0 (0.0%) | 0.130 |  |
| **SHARE (Complete-case participants: n=24,571; Covariate-incomplete participants: n=474)** | | | | | | |  |
| SHARE | Age at T0, years | 63.84 ± 8.98 | 0 (0.0%) | 63.82 ± 9.41 | 0 (0.0%) | 0.002 |  |
|  | Women, n (%) | 10,441 (42.5%) | 0 (0.0%) | 202 (42.6%) | 0 (0.0%) | 0.002 |  |
|  | Education: low, n (%) | 8,677 (35.3%) | 0 (0.0%) | 198 (41.8%) | 0 (0.0%) | 0.133 |  |
|  | Education: middle, n (%) | 9,943 (40.5%) | 0 (0.0%) | 178 (37.6%) | 0 (0.0%) | 0.060 |  |
|  | Education: high, n (%) | 5,951 (24.2%) | 0 (0.0%) | 98 (20.7%) | 0 (0.0%) | 0.085 |  |
|  | Married/partnered, n (%) | 17,266 (70.3%) | 0 (0.0%) | 41 (51.2%) | 394 (83.1%) | 0.397 |  |
|  | Self-rated health z-score | 0.00 ± 1.00 | 0 (0.0%) | -0.04 ± 0.93 | 6 (1.3%) | 0.037 |  |
|  | Depressive symptoms z-score | 0.00 ± 1.00 | 0 (0.0%) | -0.00 ± 1.06 | 0 (0.0%) | 0.004 |  |
|  | Ever smoking, n (%) | 11,447 (46.6%) | 0 (0.0%) | 89 (78.1%) | 360 (75.9%) | 0.687 |  |
|  | Current drinking, n (%) | 12,437 (50.6%) | 0 (0.0%) | 221 (52.7%) | 55 (11.6%) | 0.043 |  |
|  | Baseline subjective memory z-score | -0.13 ± 0.94 | 0 (0.0%) | 0.06 ± 0.93 | 0 (0.0%) | 0.204 |  |
|  | Baseline objective cognition z-score | 0.19 ± 0.68 | 0 (0.0%) | 0.02 ± 0.72 | 0 (0.0%) | 0.245 |  |
| **Notes:** Values are mean ± SD for continuous variables and n (%) for categorical variables. Categorical percentages are calculated among participants with nonmissing data for the corresponding characteristic; missing percentages use the total size of each comparison group. SMD values are presented as absolute standardized mean differences. An absolute SMD ≥0.10 may indicate meaningful imbalance. Outcome-ineligible participants included those with functional dependence at T0 or T1 or without a valid post-T1 outcome assessment. CHARLS, China Health and Retirement Longitudinal Study; ELSA, English Longitudinal Study of Ageing; HRS, Health and Retirement Study; SHARE, Survey of Health, Ageing and Retirement in Europe; SMD, standardized mean difference. | | | | | | |  |
|  |  |  |  |  |  |  |  |
|  |  |  |  |  |  |  |  |
|  |  |  |  |  |  |  |  |
