# Supplementary material for: Joint changes in subjective memory and objective cognition and risk of incident functional dependence: A harmonized analysis of four aging cohorts
Source: Alzheimers Dement. 2026 Aug 22;22(8):e71752. doi: 10.1002/alz.71752 (PMC13498894; doi:10.1002/alz.71752)
Supplement: Supplementary file 13 [file ALZ-22-e71752-s015.docx]

**Table S9. Multiple-imputation sensitivity analyses of the associations between joint cognitive change patterns and incident functional dependence**

| **Panel A. Cohort-specific complete-case and multiple-imputation estimates** | | | | | | | | | |
| --- | --- | --- | --- | --- | --- | --- | --- | --- | --- |
| **Cohort** | **Trajectory group** | **Complete-case N/events** | **Complete-case HR (95% CI)** | **P value** | **MI N/events** | **MI HR (95% CI)** | **P value** | **MI vs complete-case HR change, %** |  |
| CHARLS | Subjective-only decline | 14,047/3,378 | 1.257 (1.125–1.405) | <0.001 | 14,076/3,389 | 1.252 (1.120–1.399) | <0.001 | -0.5 |  |
|  | Objective-only decline |  | 1.043 (0.953–1.141) | 0.359 |  | 1.048 (0.958–1.146) | 0.308 | 0.5 |  |
|  | Concordant decline |  | 1.596 (1.397–1.824) | <0.001 |  | 1.579 (1.382–1.804) | <0.001 | -1.1 |  |
| HRS | Subjective-only decline | 19,854/6,808 | 1.142 (1.066–1.224) | <0.001 | 19,995/6,853 | 1.137 (1.061–1.219) | <0.001 | -0.5 |  |
|  | Objective-only decline |  | 1.178 (1.105–1.255) | <0.001 |  | 1.179 (1.107–1.256) | <0.001 | 0.1 |  |
|  | Concordant decline |  | 1.464 (1.335–1.605) | <0.001 |  | 1.460 (1.332–1.600) | <0.001 | -0.3 |  |
| ELSA | Subjective-only decline | 3,857/403 | 1.672 (1.280–2.183) | <0.001 | 4,591/510 | 1.637 (1.295–2.068) | <0.001 | -2.1 |  |
|  | Objective-only decline |  | 1.293 (0.985–1.697) | 0.064 |  | 1.264 (0.995–1.607) | 0.055 | -2.2 |  |
|  | Concordant decline |  | 1.531 (1.033–2.267) | 0.034 |  | 1.310 (0.910–1.885) | 0.146 | -14.4 |  |
| SHARE | Subjective-only decline | 24,571/3,548 | 1.074 (0.978–1.180) | 0.136 | 25,045/3,633 | 1.069 (0.974–1.174) | 0.160 | -0.5 |  |
|  | Objective-only decline |  | 1.077 (0.982–1.182) | 0.116 |  | 1.079 (0.984–1.182) | 0.106 | 0.1 |  |
|  | Concordant decline |  | 1.383 (1.229–1.556) | <0.001 |  | 1.407 (1.253–1.581) | <0.001 | 1.8 |  |

| **Panel B. Pooled random-effects meta-analysis of multiple-imputation estimates** | | | | | |
| --- | --- | --- | --- | --- | --- |
| **Trajectory group** | **Pooled MI HR (95% CI)** | **P value** | **I²** | **P value for heterogeneity** | **τ² (REML)** |
| Subjective-only decline | 1.223 (1.050–1.425) | 0.010 | 77.5% | 0.004 | 0.0199 |
| Objective-only decline | 1.118 (1.043–1.199) | 0.002 | 52.0% | 0.100 | 0.0024 |
| Concordant decline | 1.465 (1.376–1.559) | <0.001 | 0.0% | 0.562 | 0.0000 |

**Notes:** The concordant stable or improved group served as the reference category. Fully adjusted Cox models included baseline age, sex, education, marital status, self-rated health, depressive symptoms, smoking status, current drinking, baseline subjective memory, and baseline objective cognition. Multiple imputation was performed separately within each cohort among participants eligible for the incident functional-dependence analysis; 20 imputed datasets were generated. The imputation models included the trajectory group, all analysis covariates, the event indicator, follow-up time, and the Nelson–Aalen cumulative hazard estimate. Cohort-specific estimates were combined using Rubin’s rules and pooled using restricted maximum likelihood random-effects meta-analysis. The cohort-specific ELSA estimate for concordant decline lost statistical significance after imputation, but the direction remained positive and the pooled concordant-decline association remained robust. CI, confidence interval; HR, hazard ratio; MI, multiple imputation; REML, restricted maximum likelihood.
