# Supplementary material for: Joint changes in subjective memory and objective cognition and risk of incident functional dependence: A harmonized analysis of four aging cohorts
Source: Alzheimers Dement. 2026 Aug 22;22(8):e71752. doi: 10.1002/alz.71752 (PMC13498894; doi:10.1002/alz.71752)
Supplement: Supplementary file 14 [file ALZ-22-e71752-s016.docx]

**Table S3. Missingness of variables included in the fully adjusted models among participants eligible for the incident functional-dependence analysis**

| **Variable** | **CHARLS (N=14,076)** | **HRS (N=19,995)** | **ELSA (N=4,591)** | **SHARE (N=25,045)** |
| --- | --- | --- | --- | --- |
| Age at T0 | 7 (0.0%) | 0 (0.0%) | 0 (0.0%) | 0 (0.0%) |
| Sex at T0 | 0 (0.0%) | 0 (0.0%) | 0 (0.0%) | 0 (0.0%) |
| Education at T0 | 0 (0.0%) | 4 (0.0%) | 405 (8.8%) | 0 (0.0%) |
| Marital status at T0 | 0 (0.0%) | 6 (0.0%) | 1 (0.0%) | 394 (1.6%) |
| Self-rated health at T0 | 2 (0.0%) | 7 (0.0%) | 1 (0.0%) | 6 (0.0%) |
| Depressive symptoms at T0 | 15 (0.1%) | 10 (0.1%) | 8 (0.2%) | 0 (0.0%) |
| Ever smoking at T0 | 1 (0.0%) | 113 (0.6%) | 1 (0.0%) | 360 (1.4%) |
| Current drinking at T0 | 4 (0.0%) | 3 (0.0%) | 352 (7.7%) | 55 (0.2%) |
| Baseline subjective memory | 0 (0.0%) | 0 (0.0%) | 0 (0.0%) | 0 (0.0%) |
| Baseline objective cognition | 0 (0.0%) | 0 (0.0%) | 0 (0.0%) | 0 (0.0%) |
| **Any missing model covariate** | **29 (0.2%)** | **141 (0.7%)** | **734 (16.0%)** | **474 (1.9%)** |

**Notes:** Missingness is presented as n/N (%) among participants who met the eligibility criteria for the incident functional-dependence analysis before complete-case exclusion. Participants with missing subjective memory or objective cognition at T0 or T1 had already been excluded during trajectory derivation; therefore, baseline subjective memory and baseline objective cognition were complete in this analytic sample. “Any missing model covariate” indicates the number of unique participants with missing data for at least one variable included in the fully adjusted Cox model and should not be calculated by summing variable-specific missing counts. Multiple imputation was performed among these outcome-eligible participants. CHARLS, China Health and Retirement Longitudinal Study; ELSA, English Longitudinal Study of Ageing; HRS, Health and Retirement Study; SHARE, Survey of Health, Ageing and Retirement in Europe.
