# Supplementary material for: Joint changes in subjective memory and objective cognition and risk of incident functional dependence: A harmonized analysis of four aging cohorts
Source: Alzheimers Dement. 2026 Aug 22;22(8):e71752. doi: 10.1002/alz.71752 (PMC13498894; doi:10.1002/alz.71752)
Supplement: Supplementary file 15 [file ALZ-22-e71752-s017.docx]

**Table S5. Crude associations between joint change patterns in subjective memory and objective cognition and incident functional dependence across the four cohorts**

| **Cohort** | **Trajectory group** | **HR (95% CI)** | **P value** | **N** | **Events** |
| --- | --- | --- | --- | --- | --- |
| **CHARLS** | Subjective-only decline | 0.899 (0.816–0.989) | 0.029 | 14,047 | 3,378 |
|  | Objective-only decline | 1.033 (0.947–1.126) | 0.465 | 14,047 | 3,378 |
|  | Concordant decline | 1.136 (1.006–1.282) | 0.039 | 14,047 | 3,378 |
| **HRS** | Subjective-only decline | 1.098 (1.030–1.171) | 0.004 | 19,854 | 6,808 |
|  | Objective-only decline | 1.152 (1.083–1.225) | <0.001 | 19,854 | 6,808 |
|  | Concordant decline | 1.371 (1.257–1.497) | <0.001 | 19,854 | 6,808 |
| **ELSA** | Subjective-only decline | 1.514 (1.185–1.935) | <0.001 | 3,857 | 403 |
|  | Objective-only decline | 1.225 (0.939–1.598) | 0.134 | 3,857 | 403 |
|  | Concordant decline | 1.385 (0.954–2.010) | 0.087 | 3,857 | 403 |
| **SHARE** | Subjective-only decline | 0.971 (0.892–1.057) | 0.496 | 24,571 | 3,548 |
|  | Objective-only decline | 1.117 (1.021–1.223) | 0.016 | 24,571 | 3,548 |
|  | Concordant decline | 1.315 (1.178–1.469) | <0.001 | 24,571 | 3,548 |

**Notes:** Values are hazard ratios (HRs) and 95% confidence intervals (CIs) derived from cohort-specific unadjusted Cox proportional hazards models. The concordant stable or improved group served as the reference category. All analyses used the same final complete-case samples as the primary fully adjusted models.
